# A multimedia model to estimate the environmental fate of microplastic particles

**DOI:** 10.1016/j.scitotenv.2023.163437

**Published:** 2023-07-15

**Authors:** J.T.K. Quik, J.A.J. Meesters, A.A. Koelmans

**Affiliations:** a National Institute for Public Health and Environment (RIVM), Centre for Sustainability, Health and Environment, Antonie van Leeuwenhoeklaan 9, 3721 MA Bilthoven, the Netherlands; b National Institute for Public Health and Environment (RIVM), Centre for Safety of Substances and Products, Antonie van Leeuwenhoeklaan 9, 3721 MA Bilthoven, the Netherlands; c Aquatic Ecology and Water Quality Management Group, Wageningen University, P.O. Box 47, 6700 DD Wageningen, the Netherlands

**Keywords:** Nanoplastic, Microplastic, Multimedia model, SimpleBox, Unit world

## Abstract

Nano- and microplastic (NMP) is a diverse and challenging contaminant and data on NMP concentrations are therefore not fully available for all environmental compartments. For environmental assessments of NMP, screening-level multimedia models can fill this gap, but such models are not available. Here, we present SimpleBox4Plastic (SB4P) as the first multimedia ‘unit world’ model capable of addressing the full NMP continuum, explore its validity, and evaluate it based on a case study for microbeads and by comparisons with (limited) concentration data. SB4P links NMP transport and concentrations in and across air, surface water, sediment, and soil, taking into account processes such as attachment, aggregation, and fragmentation, by solving mass balance equations using matrix algebra. These link all concentrations and processes known to be relevant for NMP using first-order rate constants, which are obtained from the literature. The SB4P model, as applied to microbeads, provided mass or number concentrations of NMP as the total of ‘free’ particles, heteroaggregates with natural colloids, and larger natural particles in each compartment at steady state. Processes most relevant in explaining observed Predicted Exposure Concentrations (PECs) were determined using rank correlation analysis. Although the predicted PECs remained uncertain due to the propagating uncertainty, inferences regarding these processes and relative distribution across compartments can be considered robust.

## Highlights

• Nano- and microplastic (NMP) is a diverse and challenging contaminant.
• Multimedia ‘unit world’ models can simulate NMP distribution in the environment.
• SimpleBox4Plastic is the first multimedia model addressing the full NMP continuum.
• We provide a case study for microbeads and compare with measured concentrations.
• Despite propagating uncertainty, the model is robust for screening-level purposes.

## Graphical abstract

**Figure:**
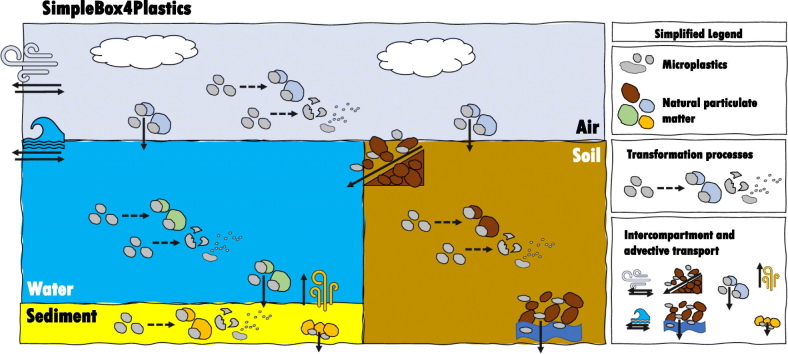

## 1 Introduction

At the moment, there is a great interest in science and society to know how plastic behaves in the environment. However, environmental plastic is not one type of material, but an extremely diverse mixture of particles of different size, shape and polymer type (Hidalgo-Ruz et al., 2012; Kooi and Koelmans, 2019; Gouin et al., 2019; Koelmans et al., 2022). Within this environmental plastic continuum, microplastic (<5 mm) small microplastics (<20 μm) and nanoplastics (<1 μm), collectively referred to as NMP, are probably the least known area of environmental litter, but possibly also the most hazardous (Shim et al., 2014; Koelmans et al., 2015). The large surface area of NMP has been shown to induce very strong sorption affinities for toxic organic chemicals (Velzeboer et al., 2014), potentially leading to cumulative particle and chemical toxicity effects once NMP have crossed cell membranes (Fournier et al., 2020). In addition to particle properties, species characteristics and the structure and dynamics of the food web can also play a role in the impact assessment (Diepens and Koelmans, 2018; Kong and Koelmans, 2019; Thornton Hampton et al., 2022). This calls for a risk assessment for these particles, which is, however, complicated because to date no methods are available to detect number concentrations and characteristics for (sub-)micron sized polymer particles in the environment (Ter Halle et al., 2017; Mintenig et al., 2018; Koelmans et al., 2022). This means that the exposure assessment for microplastics and especially the smallest and nano-fractions will have to be based on modeling for the time being, a situation that has also existed for years for non-polymeric engineered nanomaterials (Gottschalk et al., 2013; Quik et al., 2015).

Multimedia modeling is an established methodology for screening-level prospective exposure assessments for traditional contaminants such as organic micropollutants and heavy metals (Hollander et al., 2016), and also particulates such as engineered nanomaterials (Mackay and Paterson, 1991; Meesters et al., 2014; Liu et al., 2015; Meesters et al., 2016; Nowack, 2017; Meesters et al., 2019). It is based on subdividing a ‘unit-world’ into representative fractions of relevant media, such as air, surface and groundwaters, sediment, soil and biota. Such models are often used to test hypotheses, the relevance of processes, and relationships between environmental fate and pollutant characteristics. As such they can provide the first basis for quantifying exposure in the context of risk assessment (Di Guardo et al., 2018). SimpleBox is a multimedia mass balance model in which emissions, transport, and degradation of chemical substances within and between different environmental compartments are estimated (Hollander et al., 2016). The SimpleBox model is included in the European Commission's regulatory framework for the registration, evaluation, authorisation and restriction of chemicals (REACH) and forms the basis for current guidelines for predicting regional environmental background concentrations (ECHA, 2016). SimpleBox4Nano has been developed for the assessment of substances in a nanoparticle form (Meesters et al., 2014; ECHA, 2017; Quik et al., 2020). Similarly, multimedia models like the SB4N particle model can be updated and applied probabilistically, to estimate the multimedia distribution of the entire nano- and microplastic particle continuum while maintaining the continuity of the particle characteristics (Kooi et al., 2018).

Since multimedia models essentially calculate relative distributions or distribution fractions (Mackay, 2001), emission data is needed to predict environmental concentrations (PECs). For nano- and microplastics (NMP), this means identifying their sources and quantifying their source strength. For NMP, especially atmospheric sources are relevant, as they can be distributed across the main environmental compartments, while, for example, waterborne NMP is less likely to be transported to the atmosphere or directly to terrestrial systems with high fluxes (Evangeliou et al., 2020), although aerosolization at the ocean surface has been found to occur (Allen et al., 2020). This implies that especially emissions to the atmosphere seem relevant to take into account in the multimedia modeling, whereas concentrations originating from compartment-specific sources may be added to compartments afterward. For nanoplastics, we are aware of several sources of direct emission to the atmosphere, for instance, thermal cutting of plastic (Zhang et al., 2012) and 3D printing (Stephens et al., 2013; Rodriguez-Hernandez et al., 2020). Furthermore, NMP are believed to be formed by progressive fragmentation of larger items and particles until they reach the micron and sub-micron scale (Koelmans et al., 2017; SAPEA, 2019). Plastic particles have been shown to exist as a continuum of sizes, shapes and densities, with sizes ranging from <5 mm (microplastic) to the submicron nanoplastic scale (Kooi and Koelmans, 2019; Kooi et al., 2021; Mattsson et al., 2021). Nanoplastics, therefore, like any other plastic fraction, should not be considered in isolation, but are best considered in the context of this continuum in terms of their fate, exposure, impacts, and risks. However, multimedia modeling of the full continuum of sizes, including fragmentation as a source for smaller particles, is still unexplored territory (Koelmans et al., 2018).

The purpose of this article is twofold. The first and primary objective is to introduce SimpleBox4Plastic as a new multimedia model for NMP, and to evaluate the model by investigating the theoretical multimedia distributions of NMP based on NMP-specific parameters and first principles, and identifying challenges and recommendations for future work on multimedia fate modeling of NMP. The second objective is to present a case study of a preliminary calculation of PECs for microplastics parameterized as primary microplastics, hereafter referred to as microbeads, given the existing emission estimates, and work on these types of intentionally produced microplastics. This case study is particularly relevant given the REACH restriction currently being implemented for synthetic polymer microparticles (ECHA, 2019).

## 2 Methods

### 2.1 Model description

SimpleBox4Nano (version 11/03/2021) is used as a basis for a new multimedia fate model for NMP; SimpleBox4Plastic (SB4P, see Fig. S10). SimpleBox4Nano required a minor adaptation to the differential settling algorithms to account for plastics with a density smaller than water, which do not settle. Furthermore, fragmentation or degradation of microplastics has been shown to affect microplastic fate and are thus included as removal processes based on studies by (Koelmans et al., 2017; Chamas et al., 2020; Mateos-Cardenas et al., 2020; Kaandorp et al., 2021; Mattsson et al., 2021). More details on these adaptations are given in the supporting information. This new version, SimpleBox4Plastic, is available from doi: 10.5281/zenodo.7416252. The other specific aspects of microplastic behavior and fate in the environment are already covered by the nanomaterial extension of SimpleBox. This includes processes such as hetero-agglomeration of substances in the particulate form with natural particles, and particle deposition out of air to soil and water, as well as sedimentation from the water column to the sediment (Meesters et al., 2014; Meesters et al., 2016). The SimpleBox4Plastic model has been applied probabilistically using the @Risk Excell plugin (v8, Pallisade), to calculate concentrations in air, natural soil, agricultural soil, industrial soil, river water, river sediment, seawater, and marine sediment.

### 2.2 Model parametrization

#### 2.2.1 Fragmentation and degradation rate constants

The fragmentation rate constant parametrizes how fast larger plastic particles transform into smaller sizes. The available information on this process is limited, but it can be assumed that the fragmentation rates are highly variable between plastic types, shapes, and between different compartments and within compartments. Two studies that provide quantitative data for estimating the fragmentation rate constant are considered (Table S1). Both data sources apply to microplastics in the marine environment (Koelmans et al., 2017; Kaandorp et al., 2021). The resulting fragmentation rate constants range over two orders of magnitude (from 1.6 × 10^−9^ to 1.3 × 10^−7^ s^−1^) with a most likely value of 2.7 × 10^−8^ s^−1^. The fragmentation process has been modeled probabilistically, with the median and extreme parameter values based on these two studies (Table S2). Furthermore, we modeled degradation based on degradation rates provided in Chammas et al. (Tables S1 and S2). For air, it is assumed that fragmentation and degradation are negligible, due to the short residence times, even for nanoparticles (Meesters et al., 2019).

### 2.3 Emission rates and parameterization for the microbead multimedia distribution case study

A case study is performed as proof of principle of the SB4P model by simulating the multimedia distribution of microbeads based on a study by Scudo et al. (2017). This study performed an evaluation of microbeads emissions in the EU based on the European Union System for Evaluation of Substances (EUSES). The magnitude and variation of the emission rates of microbeads is inserted with a triangular distribution estimated from their low, best estimate and high release estimates during formulation and application of microbeads in different sectors, such as personal care and cosmetic products (PCCP), paints and coatings, soaps, detergents and maintenance products, abrasives, agriculture, and in the oil and gas industry (Fig. S1 and Table S4; Scudo et al. (2017)). For all these applications, the total emission volumes to the air, water, industrial and agricultural soil compartments (unit t/y) have been estimated at the regional and continental (EU) scale. The regional scale scenario is representing an industrial area. All applications have releases to water, but paints and coatings, abrasives and oil and gas industry release to soil and air. The discharges into the water are subdivided into discharges directly into the water or into the sewage system. In sewage treatment, a fraction (see Table S5) is emitted via effluent to the freshwater compartment and via sludge to agricultural land. Although the application of sludge to the soil is not done in all EU Member States, the application of soil sludge is also considered a relevant route for microbead emissions to soil, as the content of microplastics is not regulated (EC, 2022).

Microbead properties such as size and density are inserted in the model with uniform distribution ranging between the minimum and maximum size and density -as reported by Scudo et al. (2017) for different polymer types, as considered in the different applications (Table 1) so that the variation in these microbead properties is covered. The attachment efficiencies of microbeads with natural particulates (colloids or coarser particulates) can be considered highly variable due to the variability of environmental conditions such as ionic strength, pH and dissolved organic matter concentrations (Shams et al., 2020). This parameter is therefore also modeled probabilistically between 0.0001 and 1, based on a range of literature values (Table S3). Here it should be noted that the parameter ranges selected to represent microbeads are also representative for the most ecologically relevant fraction of all microplastic particles found in nature. The inserted parameter ranges cover a wide and realistic range of densities, sizes and attachment efficiencies, while fully covering the ecologically relevant size fraction in terms of bioavailability (Koelmans et al., 2020). All other model parameters where set to default values, the landscape scenario was set to EUSES defaults (EU, 2008), which excludes the lake water compartment, see Tables S6 and S7.

**Table 1:** Uniform probability distribution applied as input in SimpleBox4Plastics.

Parameter	Description	Value (range)	Source
Radius	Radius of the microbead	1–625 μm	(Scudo et al., 2017)
Density	Density of the microbead polymer	854–1302 kg/m^3^	(Scudo et al., 2017)
Alpha_het_	Attachment efficiency between microbead and natural colloid or coarse suspended particulate matter	0.0001–1	Besseling et al. (2017), Jang et al. (2022), Shams et al. (2020)

The results are given as the total particulate Predicted Environmental Concentration of microbeads (PEC_[total particulate]_; e.g. g/m^3^ or particles/m^3^). This is the sum of the three particulate species accounted for in SimpleBox4Nano: PEC_[total particulate]_ = PEC_[microbead]_ + PEC_[microbead-natural colloid]_ + PEC_[microbead-coarse particulate]_. Properties of the natural particulates in each environmental compartment are given in Table S7. Because the case study concerns microbeads, number to mass conversion assumed a spherical shape of the particles. Spearman rank correlation coefficients have been calculated to explain the relationship between PEC_[total particulate]_ and the varied input parameters as well as the sensitivity of the model output to these parameters. Ranges in concentrations described in the text relate to 50th (5th–95th) percentiles.

## 3 Results and discussion

### 3.1 Uncertainty and variability in predicted microplastic exposure concentrations

**Fig. 1:**
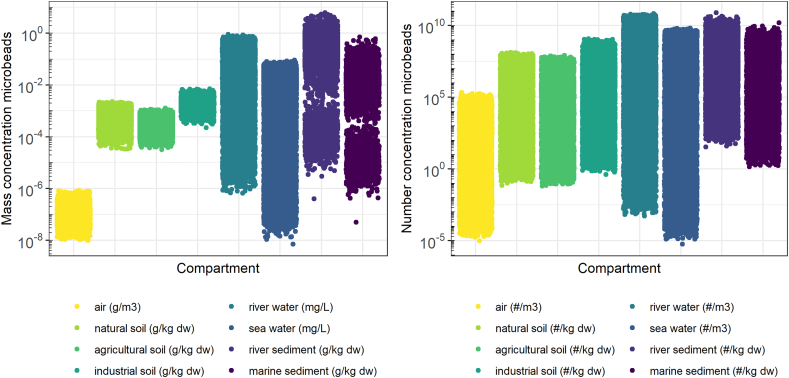
Predicted microbead Environmental Concentrations, mass based (left) and number based (right) for different compartments as calculated using SimpleBox4Plastic.

Using the probabilistic implementation of SimpleBox4Plastic, the distribution of PECs in each regional compartment is calculated (See Fig. 1). The much larger uncertainty of mass-based PECs in water and sediment, compared to the air and soil compartments stems from the added effect particle properties, such as the density, size, fragmentation, and degradation rate have on the residence time in these compartments. The uncertainty of PECs in air and soil is mainly due to uncertainty in the emission. Industrial soils have up to 5 times higher concentrations than natural and agricultural soils at the regional scale, which is to be expected because microbead emissions to industrial soils are higher (Fig. S1). Microbead concentrations in river compartments are higher than in the marine compartments, this holds for both water and sediment with up to 2 orders of magnitude difference. This can be explained by the proximity of emission sources and the lower dilution compared to seawater. Concentrations per unit dry weight in sediments are more variable than in soils, which is due to the variability in settling rates. Moreover PECs in sediments can reach a higher value. This is explained by the SB4P model simulating the sediment compartment as a converging sink for microbeads transported by run-off from large terrestrial areas to freshwater bodies with smaller surface areas. The variation in PECs can be explained by the uncertainty in emission rates and the variability and uncertainty of the particle characteristics as defined in the input parameters (Table 1). The variation in number concentrations (Fig. 1; right panel) is about 2 to 10 orders of magnitude larger than that in the mass-based concentrations (Fig. 1 left panel). This can be explained by the emission rates that initially are derived in terms of released mass per year and need conversion to emission rate as in numbers per unit of time. This conversion includes variability given the large size range of the modeled microplastics (1–625 μm). The volume is calculated from polymer density and particle size to the power of three, which explains the greater variation in number concentration. Consequently, the simulated number concentrations of microbeads in the environment comprise a highly uncertain mass-to-number conversion factor, whereas the predicted environmental mass concentrations do not. The distributions of PECs for river sediment and marine sediment are highly dependent on particle density resulting in a bimodal distribution between particle that have a lower or higher density than that of water. In the next four sections; on the four environmental compartments air, water, sediment, and soil, we further elaborate on the relative contribution of the variability and uncertainty in microplastic physicochemical properties to the variation in predicted exposure concentrations, and compare this to the variation in different emission sources of microbeads.

### 3.2 Air

**Fig. 2:**
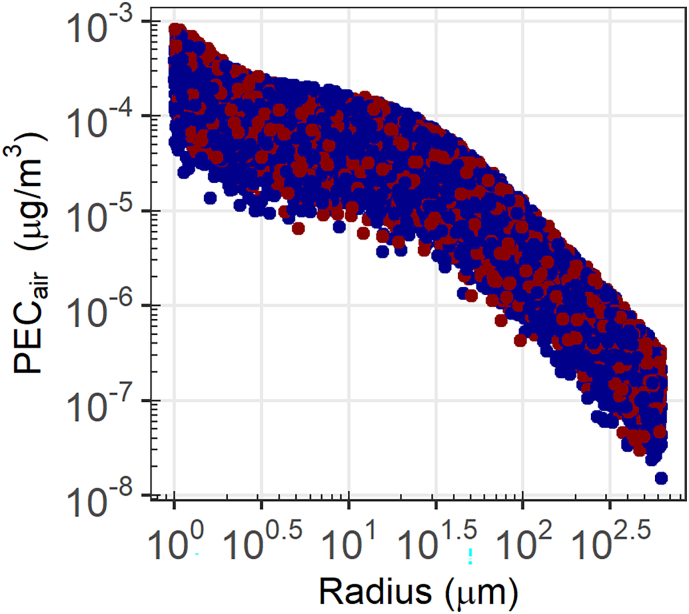
Log-log plot of PEC_dry air_ vs microbead particle radius. The red and blue datapoints correspond to particles with density below and above that of water, respectively.

The fate of microplastics in air is mainly dependent on particle size, although the largest variation is due to uncertainty in the emission estimates. Therefore, PEC_air_ is largely explained by the uncertainty in the rate of release to air from the use of microbeads as abrasives (Spearman rank coefficient 0.92). However, it appears that the size of microbeads also has an effect (Spearman rank coefficient −0.36) on the regional PEC_air_ (Figs. 2 and S3), with the negative coefficient implying that PEC_air_ is larger for smaller particles. Moreover, SB4P simulates that larger particles are more effectively removed from the atmosphere by dry deposition (Fig. S3B).

### 3.3 Freshwater and seawater

**Fig. 3:**
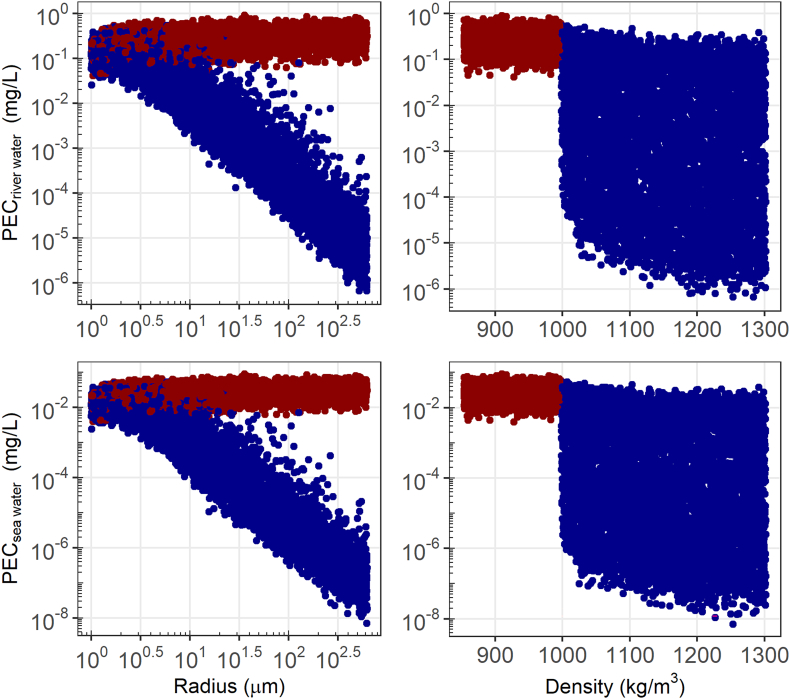
Log-log plot of PEC_fresh water_ (top) and PEC_sea water_ (bottom) versus microbead radius (left) and density (right). The red and blue datapoints correspond to particles with density below and above that of water, respectively.

**Fig. 4:**
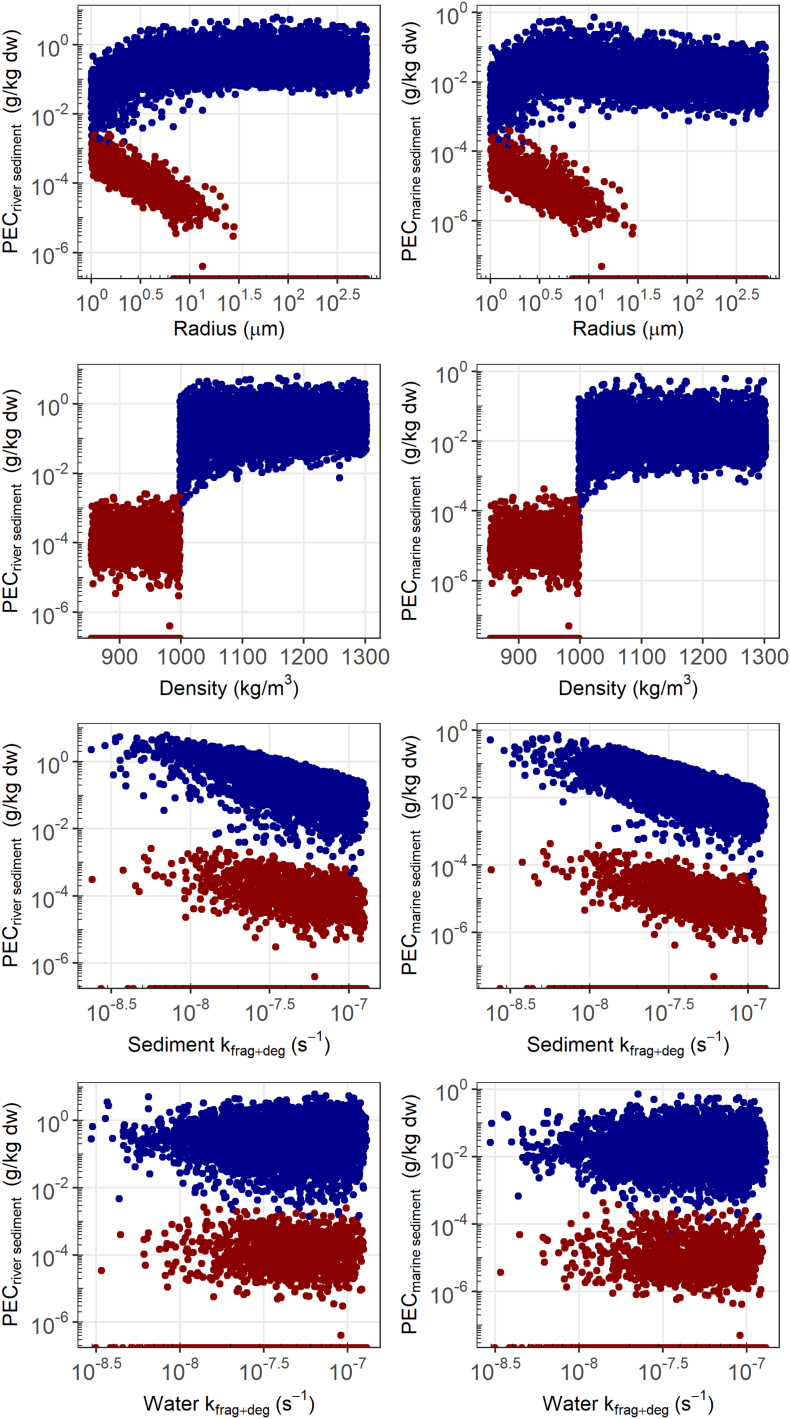
Log-log plot of PEC_sediment_ river (left) and marine (right) at regional scale plotted against microbead particle radius, density (linear scale) and the fragmentation rate constants for sediment and water (k_frag+deg_). The red and blue datapoints correspond to particles with density below and above that of water, respectively. In the top row, only low density microbeads (red points) smaller than about 10 μm enter the sediment due to heteroagglomeration with SPM. The data points on the x-axis relate to a concentration of zero.

The fate of microplastics in water is mainly dependent on particle density and size as these influence the rate of sedimentation in SB4P. Other relevant fate processes are inputs due to run-off and erosion from soil and the heteroagglomeration of microplastics with suspended particulate matter (SPM). As such, the variation in PEC_fresh water_ (Figs. 3 and S4) is mainly explained by particle density (Spearman rank coefficient 0.68), size (Spearman rank coefficient 0.43), release from use as an abrasive (Spearman rank coefficient 0.19), and the fragmentation rate constant in soil (Spearman rank coefficient 0.10). The variation in regional PEC_sea water_ is explained by the same input parameters with only minor differences (Fig. 3). Density clearly affects PECs as it separates the floating particles from the settleable particles (Fig. 4). In case the fragmentation and degradation rate constant would be set to zero, the PEC_sea water_ would increase by 10 orders of magnitude for low-density beads (Fig. S9), because this is the only parameterized removal process for microbeads that do not settle from the water column. This means that if degradation rates are extremely low other removal processes become more important to include. In principle, advection would remove lightweight microbeads consecutively from regional to continental to global scale waters, but because these scales are connected through ocean currents, the concentration continues to increase over time. It would take more than a million years to reach steady state (Figs. S7 and S8). Sedimentation of lightweight microbeads only occurs when they are less than about 10 μm because the heteroagglomerates formed by agglomeration of microbeads and SPM have a combined density dominated by that of SPM, which is higher than that of water, see Fig. 4 (Besseling et al., 2017). The steady state PEC reported in the supporting information (Fig. S9) without fragmentation is thus artificial and not realistic because other removal processes become relevant at such long timescales. Such other removal processes that might be relevant are bio/chemical degradation due to feeding (Mateos-Cardenas et al., 2020) or sea spray aerosolization and consequent transport to land (Allen et al., 2020).

### 3.4 Sediment

The fate of microplastics in sediments is mainly dependent on the microplastic density and the fragmentation and degradation rate as these affect the input of microplastics due to sedimentation rate and the removal, respectively. Thus, the variation in the regional PEC_sediment_ is dependent on the variability of the microbead density (Spearman rank coefficient 0.69), uncertainty and variability in the fragmentation rate constant for soil (Spearman rank coefficient 0.32), on release from use of polymer based abbrasives (Spearman rank coefficient 0.20), and on microbead size (Spearman rank coefficient 0.10) (Fig. 4). Remarkably, for high density microbeads only a small effect of size on the PEC_sediment_ (Spearman rank coefficient ~0.1) is seen, but for low density microbeads the effect of microbead size is much larger (Spearman rank coefficient 0.79) with a sharp cutoff at about 10 μm, above which no microbeads are transported to the sediment (see first column of panels in Fig. 4). This can be explained by the relative increase in density when a microbead attaches to an SPM particle due to heteroagglomeration (Besseling et al., 2017). When the combined density is larger than that that of water, the SPM-microbead heteroaggregate loses its buoyancy and settles to the sediment. The cut-off at about 10 μm means that the currently simulated SPM (Radius 3 μm, Density: 2500 kg/m^3^), is too light to increase the heteroaggregate density above that of water for microbeads of larger size.

The slight increase of PEC_sediment_ with microbead size up to about 5 μm can be explained by the changes in the ratio of SPM to microbead number concentration (Fig. 4). As a constant emission in mass of microbeads is assumed, the resulting number concentration increases with decreasing microbead radius. As the SPM concentration is considered constant, this results in less SPM-microbead heteroaggregates at lower microbead sizes (for high density polymer microbeads), which facilitate sedimentation. For low density polymer microbeads this effect is not observed because it is smaller than the effect of buoyancy of low density microbeads.

### 3.5 Soil

**Fig. 5:**
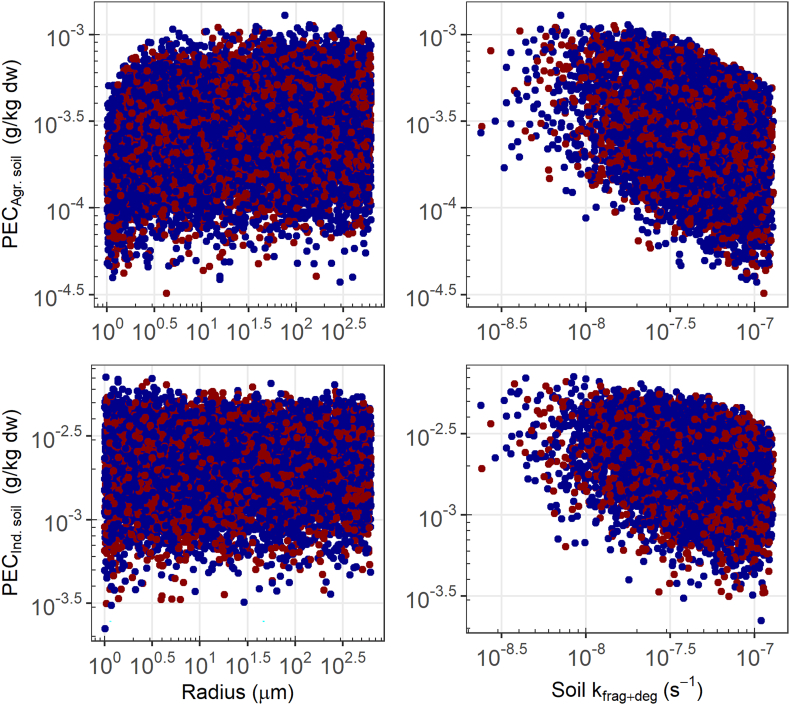
Log-log plot of microbead PEC_agricultural soil_ (top) and PEC_industrial soil_ (bottom) versus particle radius (left) and versus the fragmentation and degradation rate constant (right). The red and blue datapoints correspond to particles with densities lower and higher than that of water, respectively.

The fate of microplastics in the soil is largely dependent on the degradation and fragmentation rate, although the main variability in the PEC_soil_ is due to uncertainty in the emission estimates (Fig. 5). There are some differences regarding which emission source is most relevant (Spearman rank coefficient 0.11–0.86). Additionally, the uncertainty in the fragmentation and degradation rate constant also explains part of the variation in PEC_soil_ (Spearman rank coefficient 0.16–0.33). Variation in industrial soil PECs are mostly related to direct emission from the application as abrasive (Spearman rank coefficient 0.86) and to a small degree application in the oil industry (Spearman rank coefficient 0.11). The variation of industrial soil PECs is also related to uncertainty in the release to air from use as abrasive (industrial Spearman rank coefficient 0.35). The uncertainty for this route of release explains almost all of the variation of PECs in agricultural and natural soil (agriculture Spearman rank coefficient 0.87, natural soil Spearman rank coefficient 0.93) at the regional scale. For these two soil types, the variation in PEC is also correlated slightly to the microbead radius (natural soil Spearman rank coefficient 0.23, and agricultural soil Spearman rank coefficient 0.21). This is due to the emission scenario at the regional scale representing an industrial area with a much lower fraction of agricultural use. At the European scale, the emission scenario is more balanced between industrial, agricultural, and urban use of microbeads. At this scale, the uncertainty in release due to application in agriculture explains the highest variation in PEC_agricultural soil_ (Spearman rank coefficient 0.62) and to a lesser extent the uncertainty in STP efficiency (Spearman rank coefficient 0.11) and the microbead applications contributing to this emission route (Spearman rank coefficient 0.32 for PCCP use). See Fig. S2b for EU scale PECs.

### 3.6 Comparison of modeled and measured data

No data is available to validate the modeled multimedia distribution of microplastic beads. Concentration data are available for aquatic compartments such as rivers and surface waters of the ocean, but hardly for soils and not for the air compartment. In addition, the emissions underlying the microbeads case study carry significant uncertainty and do not apply to the broader class of microplastics found in nature. However, just as similar illustrative comparisons have been made for engineered nanomaterials (e.g. Gottschalk et al., 2013), we can also provide such an illustrative example here. A more detailed analysis of empirical multimedia data for heterogeneous microplastic mixtures is then warranted and urgent once such emission and environmental abundance data become available.

**Fig. 6:**
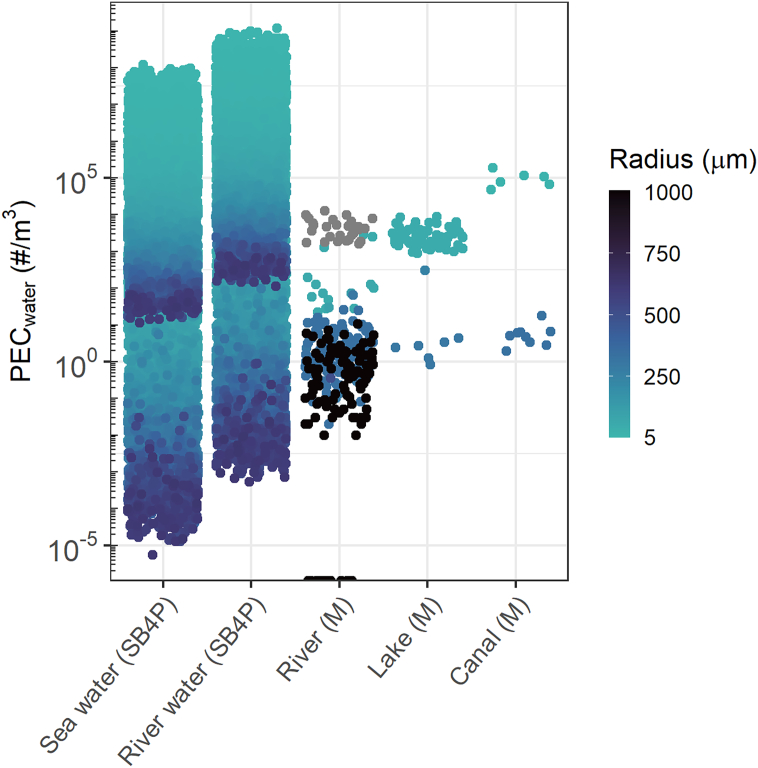
Comparison of modeled particle concentrations in river water and sea water (three bands on left; present study) with measured concentrations (data summary from Koelmans et al. (2019)) for river water, lake water and canals. Colour follows the minimum size measured in a gradient from 5 (green) to 1000 μm (black). Grey measurements did not report minimum size.

Here we provide a screening level comparison for the modeled freshwater (river) compartment and measured microplastics in three different aquatic compartments with unit particles (#) per m^3^ (Fig. 6). As mentioned in relation to Fig. 1, the modeled number concentrations vary by >8 orders of magnitude, with the modeled lower concentrations referring to the larger particles (i.e., up to 1000 μm). Given such wide spreads and lack of compatibility of the modeled and measured particle types, we do not claim significance here in terms of validation, but it is positive that the data does not contradict empirical data, that is, those data are located not outside the extremes of the modeled distributions. Here, the only exception is a handful of observations that did not detect microplastics at all (dots on the x-axis in Fig. 6), which are most likely due to analytical limitations such as too small a sample volume or too high a detection limit for particle size. Notable is the modeled trend that higher particle number concentrations are detected for smaller particles compared to larger particles, similar to the observations. Here the limit of detection also plays a role, and we set the limit of PEC_water_ estimates here at a radius of 5 μm. Lower number concentrations are observed when a higher limit of detection, e.g. 50 μm, is used as cutoff (see Fig. S2). In future work, we intend to use empirical soil, sediment and seawater concentration data to validate the model output, with an emphasis on relative ratios of the concentrations, as relevant for a screening level model such as SimpleBox.

## 4 General discussion and outlook

We provided a first parameterization using a multimedia model for microbeads. Although comparison of local measurements with predictions of regional background concentrations as predicted with SimpleBox is not straightforward, simulation results are consistent with current knowledge and design criteria. It is noted that the comparison with empirical data does not solely apply to emissions of microbeads, but relates to more sources of microplastics. A broader comparison will be provided in follow up work, as soon as sufficient high quality data is available, especially for soils. A key merit of screening-level multimedia models is their ability to rank the relevance of processes and parameters in explaining the PECs for the various compartments, like we also demonstrated here. We note that the significance of the processes and model parameters, as well as relative distribution across compartments, is independent of the emissions. This implies that these inferences apply to microplastic in general, as long as their characteristics remain sufficiently close to the modeled parameter ranges (Table 1). As mentioned, this was the case which implies that apart from the predicted PECs, all conclusions from the present study also apply to microplastic particles in general. Finally, we recommend including fragmentation as a parameter directly affecting particle size distribution over time to improve predictions of microplastic concentrations. To this end, degradation and fragmentation constants, even if small, should be studied more closely, as even small changes can have a large impact on the build-up of microplastics in the environment. In connection with this model, it may be important to include studies of other fate processes such as sea spray or beaching of floating microplastics, as well as the effects of particle shape. Also biota have an effect on fate and transport processes of microplastics, such as degradation, fragmentation or deposition. However, no quantitative relationships are currently available to implement these. Furthermore, the application of size, shape and density distributions (Probability Density Functions; Koelmans et al., 2022) of microplastics that are representative of their application, emission route, or the compartments in which they occur can lead to further improvement of the results.

## CRediT authorship contribution statement

**J.T.K. Quik:** Conceptualization, Formal analysis, Funding acquisition, Investigation, Methodology, Software, Visualization, Writing – original draft, Writing – review & editing. **J.A.J. Meesters:** Conceptualization, Visualization, Writing – review & editing. **A.A. Koelmans:** Conceptualization, Formal analysis, Funding acquisition, Investigation, Methodology, Writing – original draft, Writing – review & editing.

## Declaration of competing interest

The authors declare that they have no known competing financial interests or personal relationships that could have appeared to influence the work reported in this paper.

## Data Availability

The link to the model repository is provided and input data is reported in the supporting information.

## Appendix A Supplementary data

Tables with model input parameters; Description of SimpleBox adaptations; Additional figures Image 1
