# Supplementary material for: A multimedia model to estimate the environmental fate of microplastic particles
Source: Sci Total Environ. 2023 Jul 15;882:163437. doi: 10.1016/j.scitotenv.2023.163437 (PMC10238883; doi:10.1016/j.scitotenv.2023.163437)
Supplement: Supplementary file 1 — Tables with model input parameters; Description of SimpleBox adaptations; Additional figures [file mmc1.docx]

**Supporting Information**

**A multimedia model to estimate environmental fate of microplastic particles**

J.T.K. Quik, J. Meesters, A.A. Koelmans

Table S1: Literature sources of fragmentation and degradation rate constants.

| **Source** | **Min (s^-1^)** | **Most likely (s^-1^)** | **Max (s^-1^)** | **Method of derivation of rate constant k (s^-1^)** |
| --- | --- | --- | --- | --- |
| (Koelmans et al., 2017)  Marine – fragmentation | 3.3E-9 | 9.3E-9 | 1.3E-7 | Based on distribution of fragmentation rate per surface area (1.4E-7 m2/year (5E-8 – 2E-6)) and surface area of plastic available for degradation of 2.07E6 m^2^. |
| (Kaandorp et al., 2021)  Marine – fragmentation | 1.6E-9 | 2.7E-8 | 8.1E-8 | Based on their assumption of the fragmentation rate of 1.8E-2 – 3.9E-2 f/year with fragmentation index (f) ranging from 0.027 – 0.36. |
| (Chamas et al., 2020) marine - degradation | 0 | 1E-10 | 2E-9 | Fit of first-order rate constant to zeroth order specific surface degradation rate of microbeads as reported (radius 8.8 mm, density: 950 kg/m3, m0: 2.75 g and SSDR ranging from 1 µm/year to 1400 µm/year) |
| (Chamas et al., 2020)  soil - degradation | 0 | 3E-11 | 1E-9 | Fit of first-order rate constant to zeroth order specific surface degradation rate of microbeads as reported (radius 8.8 mm, density: 950 kg/m3, m0: 2.75 g and SSDR ranging from 1 µm/year to 1400 µm/year) |

Table S2: Fragmentation and degradation rate constants (s-1) as applied for different compartments using a triangular distribution for the free (S), aggregated (A) and attached (P) particle species.

| **Rate constant** | **Source** | **Min (s^-1^)** | **Average (s^-1^)** | **Max (s^-1^)** | **Method of extrapolation** |
| --- | --- | --- | --- | --- | --- |
| k_frag-air_ | - | - | 0 | - |  |
| k_mpdeg-air_ | - | - | 0 | - |  |
| k_frag-water_ (applied to sea (w2) and fresh water (w0,w1)) | Koelmans 2017 and Kaandorp 2021 | 1.6E-9 | 2.7E-8 | 1.3E-7 | Min and most likely value based on Kaandorp study. Max based on estimate from Koelmans 2017.  Assumed no fragmentation in deep sea water. |
| K_mpdeg-water_  (applied to surface (w2) and deep sea (w3) and fresh water (w0, w1)) | Chamas 2021 | 1e-20 | 1E-10 | 2E-9 | Fit of first order rate constant to zeroth order specific surface degradation rate of microbeads as reported (radius 8.8 mm, density: 950 kg/m3, m0: 2.75 g and SSDR ranging from 1 µm/year to 1400 µm/year) |
| K_frag-soil_ (applied to all)  &  K_frag-sediment_ (applied to all) | Koelmans 2017 and Kaandorp 2021 | 1e-20 | 2.7E-8 | 1.3E-7 | As soil can be less dynamic in terms of mixing and shear compared to surface sea water, and the lack of information (large uncertainty) the minimum fragmentation rate constant is set to near-zero: 1e-20 s^-1^ |
| K_mpdeg-soil_ (all)  K_mpdeg-sediment_ (all) | Chamas 2021 | 1e-20 | 3E-11 | 1E-9 | Fit of first order rate constant to zeroth order specific surface degradation rate of microbeads as reported (radius 8.8 mm, density: 950 kg/m3, m0: 2.75 g and SSDR ranging from 1 µm/year to 1400 µm/year) |

Table S3: Literature sources for the attachment efficiency for heteroaggregation

| **Parameter** | **Description** | **Value (range)** | **Source** |
| --- | --- | --- | --- |
| Alpha_het_ | Attachment efficiency between microbead and natural colloid or coarse suspended particulate | 0.004 – 0.2 | Besseling et al. (2017) |
|  |  | 0.14 – 0.4 | Jang et al. (2022) |
|  |  | 0.001 - 1 | Shams et al. (2020) |


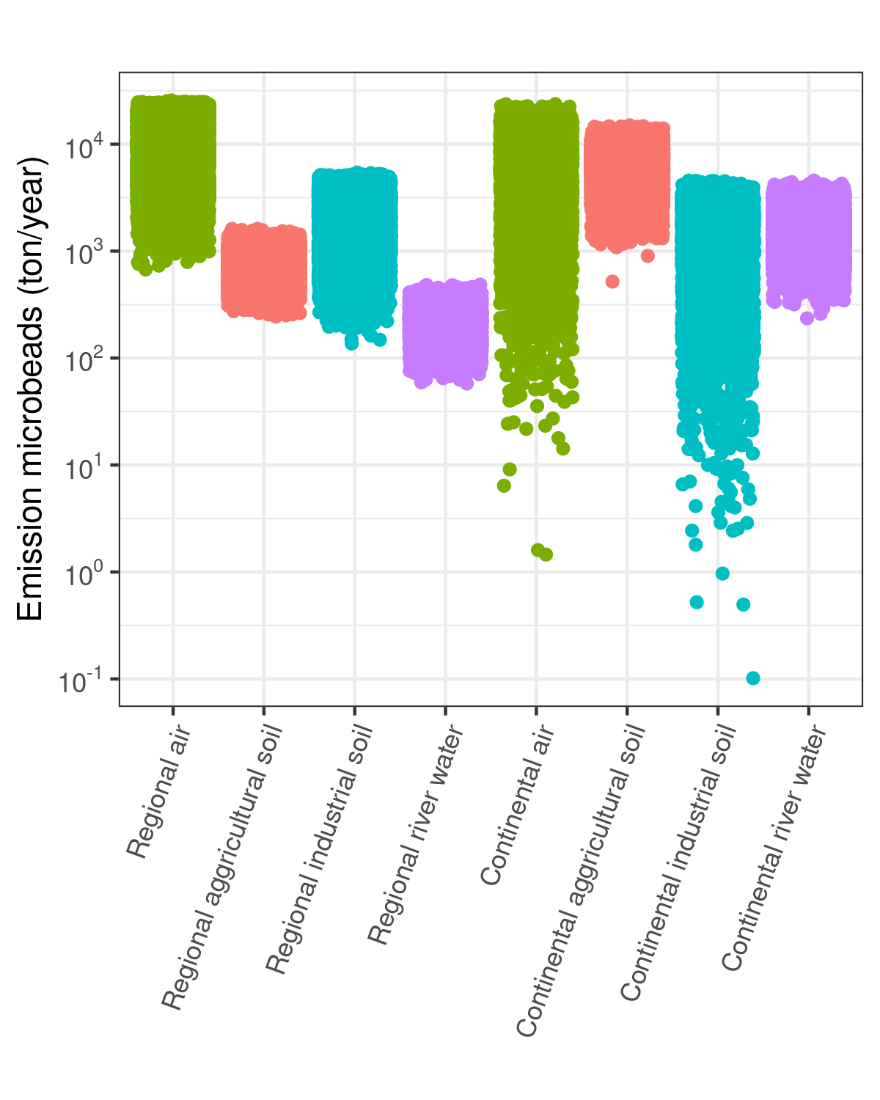


Figure S1. Scatter plot of emission rates to different compartments as calculated from regional and EU release estimates to different compartments based on (Scudo et al., 2017).

Table S4: Overview of microplastics released to air, water and industrial or agricultural soil. Discharges to water include direct discharge and discharge to sewage, thus before the sewage treatment plant (STP). from applying intentionally produced polymer microplastics in Personal Care and Cosmetics Products (PCCP), Paints and coatings, Soaps detergents and maintenance products, abrasives, Oil and gas industry and agriculture based on (Scudo et al., 2017). The regional scale represents an industrial area.

|  | | **Regional scale** | | | **EU scale** | | |
| --- | --- | --- | --- | --- | --- | --- | --- |
| PCCP |  | **NMP releas (kg/day)** | | | **NMP releas (kg/day)** | | |
| FORMULATION | | low | b.e.^a^ | high | low | b.e. | high |
|  | **Water (before STP)** | 4.6 | 4.8 | 6.7 | 46 | 48 | 300 |
|  | USE |  |  |  |  |  |  |
|  | **Water (before STP)** | 344 | 361 | 502 | 3440 | 3610 | 22500 |
| PAINTS COATINGS | |  |  |  |  |  |  |
| FORMULATION | | low | b.e. | high | low | b.e. | high |
|  | **Air** | 0.58 | 1 | 1 | 5.8 | 10 | 10 |
|  | **Water (before STP)** | 0.3 | 0.52 | 0.52 | 3 | 5.2 | 5.2 |
|  | USE |  |  |  |  |  |  |
|  | **Air** | 66.3 | 115 | 115 | 663 | 1150 | 1150 |
|  | **Water (before STP)** | 60.3 | 104 | 104 | 603 | 1040 | 1040 |
|  | **Soil** | 15.1 | 26 | 26 | 151 | 260 | 260 |
| SOAPS detergents and maintenance products | |  |  |  |  |  |  |
| FORMULATION | | low | b.e. | high | low | b.e. | high |
|  | **Water (before STP)** | 0.066 | 0.66 | 6.6 | 0.164 | 1.64 | 16.4 |
|  | USE |  |  |  |  |  |  |
|  | **Water (before STP)** | 5.48 | 54.8 | 548 | 54.8 | 548 | 5480 |
| ABRASIVES |  |  |  |  |  |  |  |
| FORMULATION | | low | b.e. | high | low | b.e. | high |
|  | **Air** | 1.37 | 13.7 | 137 | 1.37 | 13.7 | 137 |
|  | **Water (before STP)** | 1.37 | 13.7 | 137 | 1.37 | 13.7 | 137 |
|  | **Industrial Soil** | 0.68 | 6.8 | 68 | 0.68 | 6.8 | 68 |
|  | USE |  |  |  |  |  |  |
|  | **Air** | 685 | 6850 | 68500 | 685 | 6850 | 68500 |
|  | **Industrial Soil** | 137 | 1370 | 13700 | 137 | 1370 | 13700 |
| OIL and GAS industry | |  |  |  |  |  |  |
| FORMULATION | | low | b.e. | high | low | b.e. | high |
|  | **Air** | 6.85 | 68.5 | 685 | 6.85 | 68.5 | 685 |
|  | **Water (before STP)** | 5.48 | 54.8 | 548 | 5.48 | 54.8 | 548 |
|  | **Industrial Soil** | 0.027 | 0.27 | 2.7 | 0.027 | 0.27 | 2.7 |
|  | USE |  |  |  |  |  |  |
|  | **Air** | 13.7 | 137 | 1370 | 13.7 | 137 | 1370 |
|  | **Water (before STP)** | 13.7 | 137 | 1370 | 13.7 | 137 | 1370 |
|  | **Industrial Soil** | 13.7 | 137 | 1370 | 13.7 | 137 | 1370 |
| AGRICULTURE | |  |  |  |  |  |  |
| FORMULATION | | low | b.e. | high | low | b.e. | high |
|  | **Air** | 6.85 | 68.5 | 685 | 6.85 | 68.5 | 685 |
|  | **Water (before STP)** | 5.48 | 54.8 | 548 | 5.48 | 54.8 | 548 |
|  | **Industrial Soil** | 0.027 | 0.27 | 2.7 | 0.027 | 0.27 | 2.7 |
|  | USE |  |  |  |  |  |  |
|  | **Water (direct)** | 1.37 | 13.7 | 137 | 13.7 | 137 | 1370 |
|  | **Aggricultural Soil** | 26 | 260 | 2600 | 260 | 2600 | 26000 |

a: Beste Estimate for top of triangular distribution.

**Table S5:** Fraction release from water at the sewage treatment to fresh water or sludge, which is then deposited on agricultural soil. Release from the sewage treatment plant is calculated using the mass flow in water prior to sewage treatment (See table S4). This is done using a uniform distribution of the release fraction, to water or to sludge, respectively.

|  |  | **Regional scale** | | | **EU scale** | | |
| --- | --- | --- | --- | --- | --- | --- | --- |
| For emission from STP: | | min | u. | max | min | u. | max |
|  | **To water** | 0.16 |  | 0.47 | 0.16 |  | 0.47 |
|  | **To sludge and agricultural soil^a^** | 0.53 |  | 0.84 | 0.53 |  | 0.84 |

**Table S6:** Landscape setting modified from the SimpleBox4.0 defaults to resemble the EUSES default scenario. This scenario does not include the lake water compartment.

|  |  | **Regional scale** | | | **EU scale** | | |
| --- | --- | --- | --- | --- | --- | --- | --- |
|  | | value |  |  | value |  | Unit |
|  | **Area land** | 4.00E4 |  |  | 3.52E6 |  | km^2^ |
|  | **Area sea** | 4.00E2 |  |  | 3.52E6 |  | km^2^ |
|  | **Fraction lake water** | 1.00E-10 |  |  | 1.00E-10 |  | (-) |
|  | **Fraction fresh water** | 0.03 |  |  | 0.03 |  | (-) |

**Table S7:** Default SimpleBox4nano properties of environmental particulates

| ENVIRONMENTAL PROPERTIES (ALL SCALES) | | SimpleBox variable | value | unit |
| --- | --- | --- | --- | --- |
|  | Radius nucleation mode aerosol particle | RadNuc | 1.00E+01 | [nm] |
|  | Density nucleation mode aerosol particle | RhoNuc | 1.30E+03 | [kg.m-3] |
|  | Radius accumulation mode aerosol particle | RadAcc | 5.80E+01 | [nm] |
|  | Density accumulation mode aerosol particle | RhoAcc | 2.00E+03 | [kg.m-3] |
|  | Radius coarse mode aerosol particle | RadCP.a | 9.00E-01 | [µm] |
|  | Density Coarse mode aerosol particle | RhoCP.a | 2.00E+03 | [kg.m-3] |
|  | Radius natural colloids (NC, < 450 nm) in water | RadNC.w | 1.50E+02 | [nm] |
|  | Density natural colloids (NC, < 450 nm) in water | RhoNC.w | 2.00E+03 | [kg.m-3] |
|  | Radius natural suspended particulate matter (SPM, > 450 nm) in water | RadSPM.w | 3.00E+00 | [µm] |
|  | Density natural suspended particulate matter (SPM, > 450 nm) in water | RhoSPM.w | 2.50E+03 | [kg.m-3] |
|  | Radius natural colloids (NC, < 450 nm) in sediment pore water | RadNC.sd | 1.50E+02 | [nm] |
|  | Density natural colloids (NC, < 450 nm) in sediment pore water | RhoNC.sd | 2.00E+03 | [kg.m-3] |
|  | Radius filtration particles in sediment (FP, >450 nm) | RadFP.sd | 1.28E+02 | [µm] |
|  | Radius natural colloids (NC, < 450 nm) in soil pore water | RadNC.s | 1.50E+02 | [nm] |
|  | Density natural colloids (NC, < 450 nm) in soil pore water | RhoNC.s | 2.00E+03 | [kg.m-3] |
|  | Radius filtration particulates in soil (FP, > 450 nm) | RadFP.s | 1.28E+02 | [µm] |
|  | Density filtration particulates in soil (FP, > 450 nm) | RhoFP.s | 2.50E+03 | [kg.m-3] |

# SimpleBox4plastic adaptations from SimpleBox4nano

SimpleBox4plastic is based on SimpleBox4nano as described in Meesters et al. (2014). This release has minor adaptions described below.

## Density

Any function for variables dependent on the density of NMP needed to be adjusted in order to cope with densities smaller than the density of water. This was needed for the processes of agglomeration and sedimentation. The adjustments where made by including the absolute of the difference between the density of water and that of the particle suspended in it, for instance see the equations below. However, for sedimentation the sedimentation rate constants defaults to 10^-20^ s^-1^ in case a value below 0 was calculated.

$$f_{grav(i,j)}=\pi\left( r_{i}+r_{j} \right)^{2}\cdot\left| v_{set(i)}-v_{set(j)} \right|$$

Where

$$v_{set(i)}=\frac{2 \left( \rho_{i}-\rho_{water} \right)g {r_{i}}^{2}}{9\mu_{water}}$$

f_grav_ (s^-1^) is the collision frequency due to gravitational settling differences

v_set(i)_ (m.s^-1^) is gravitational settling velocity of suspended particle*.*

r (m) is the particle radius

g (m.s^-2^) is the gravitational acceleration

ρ (kg.m^-3^) is density of particle or water

µ_water_ (Pa.s) is the dynamic viscosity of water

## Degradation

Degradation is the process of mineralization of polymers. Fragmentation is a separate process of polymer particles falling apart in smaller particles due to environmental processes. Both of these processes are assumed to transform the investigated NMP into a form not taken into account anymore. In future work the mass flow due to fragmentation should affect the probability distribution of NMP size over time. Dissolution is the process of transformation from the particulate to the defined molecular species. This can be used to simulate leaching of a plastic additive (not shown here).


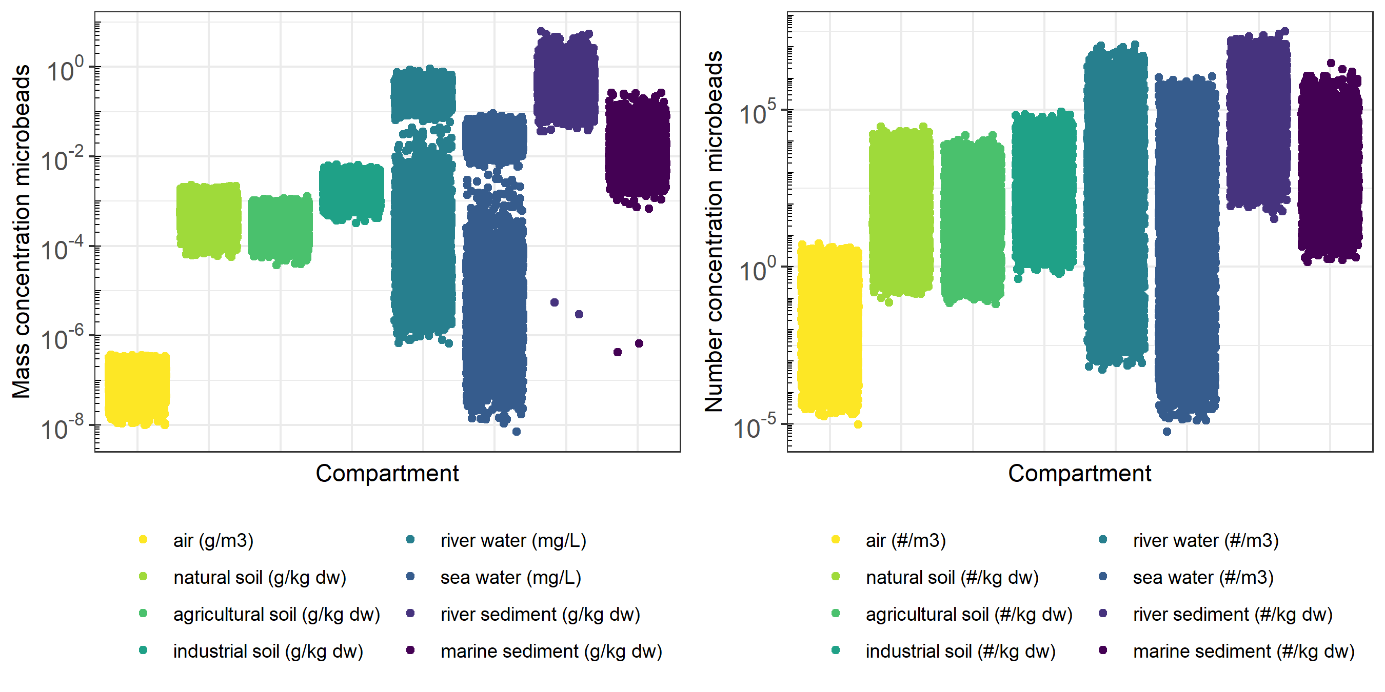


**A**

**B**


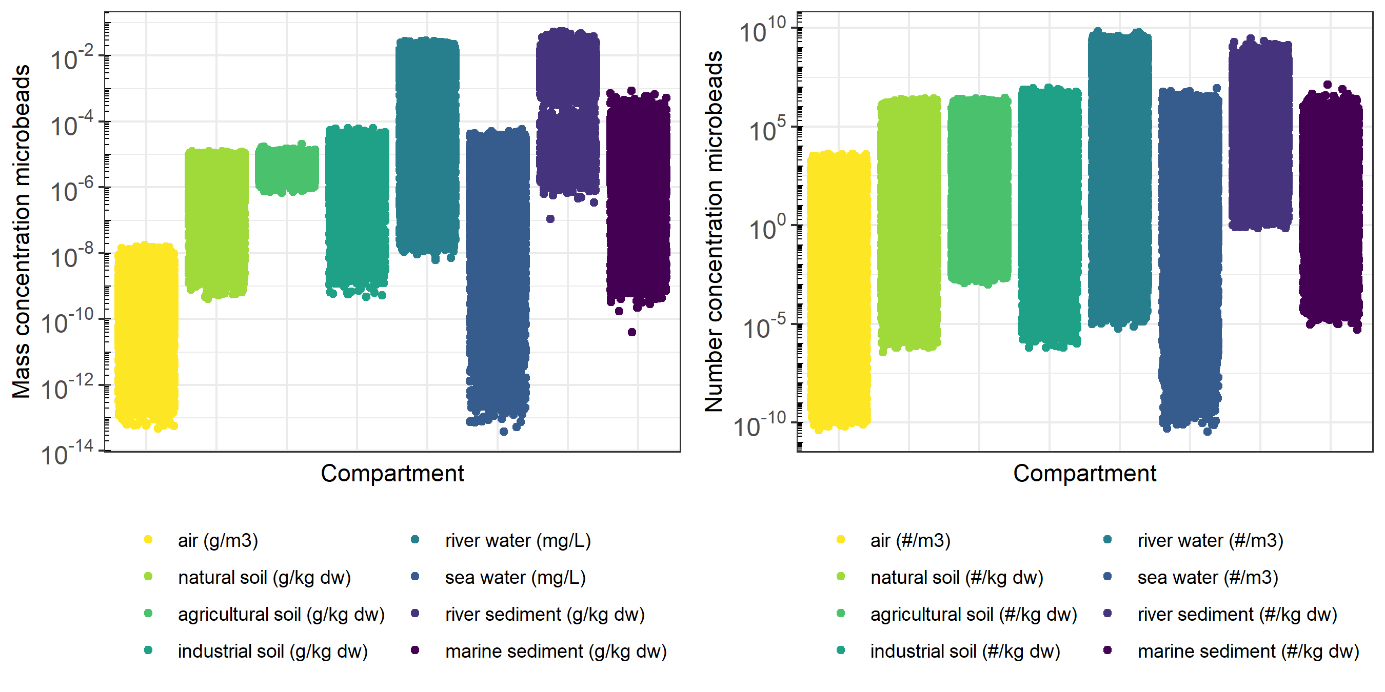


Figure S2: Predicted Environmental Concentrations (mass (left) and number based (right) for different compartments as calculated using SimpleBox4Plastics at Regional Scale (panel A), for particles larger than 50 um in diameter. PECs for all sizes at EU (continental) scale (panel B).

**B**

**A**


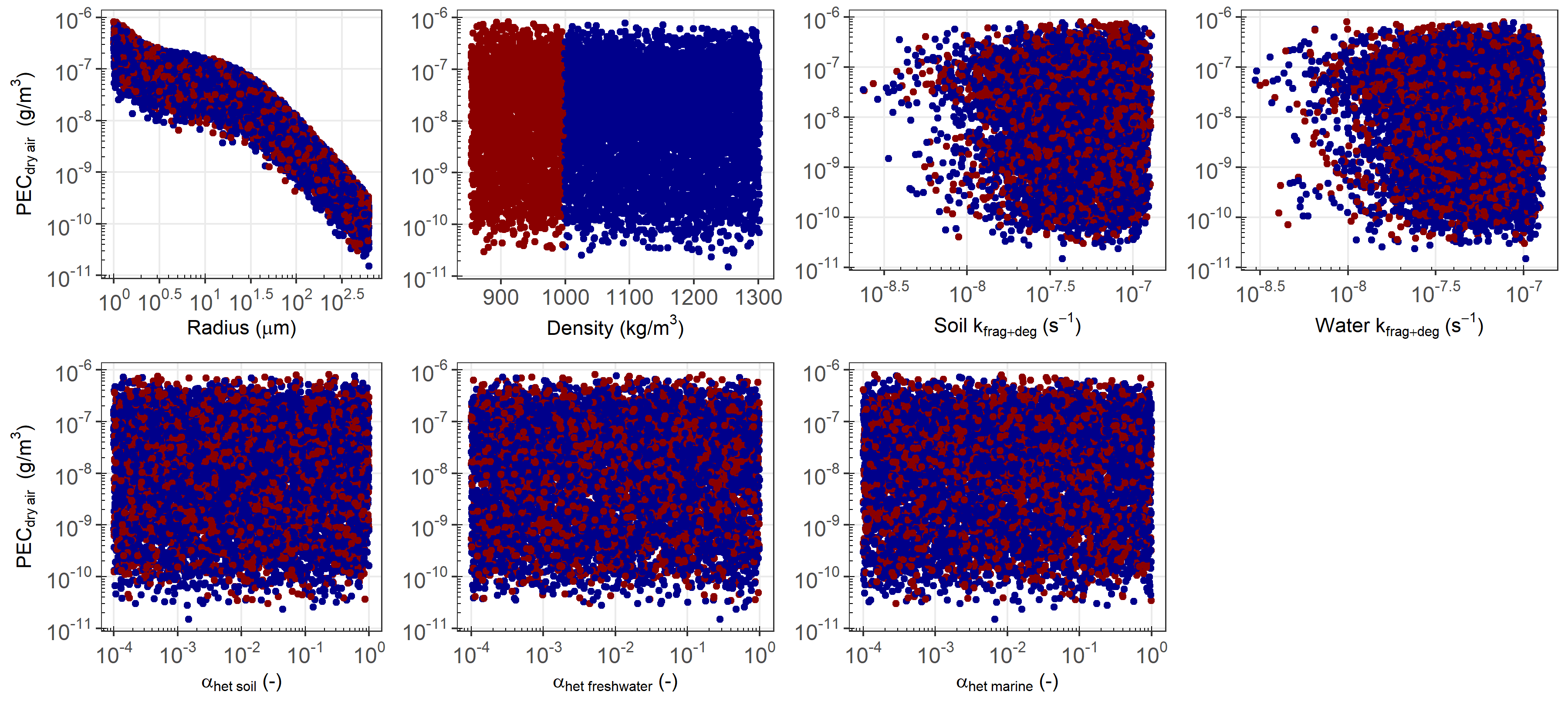


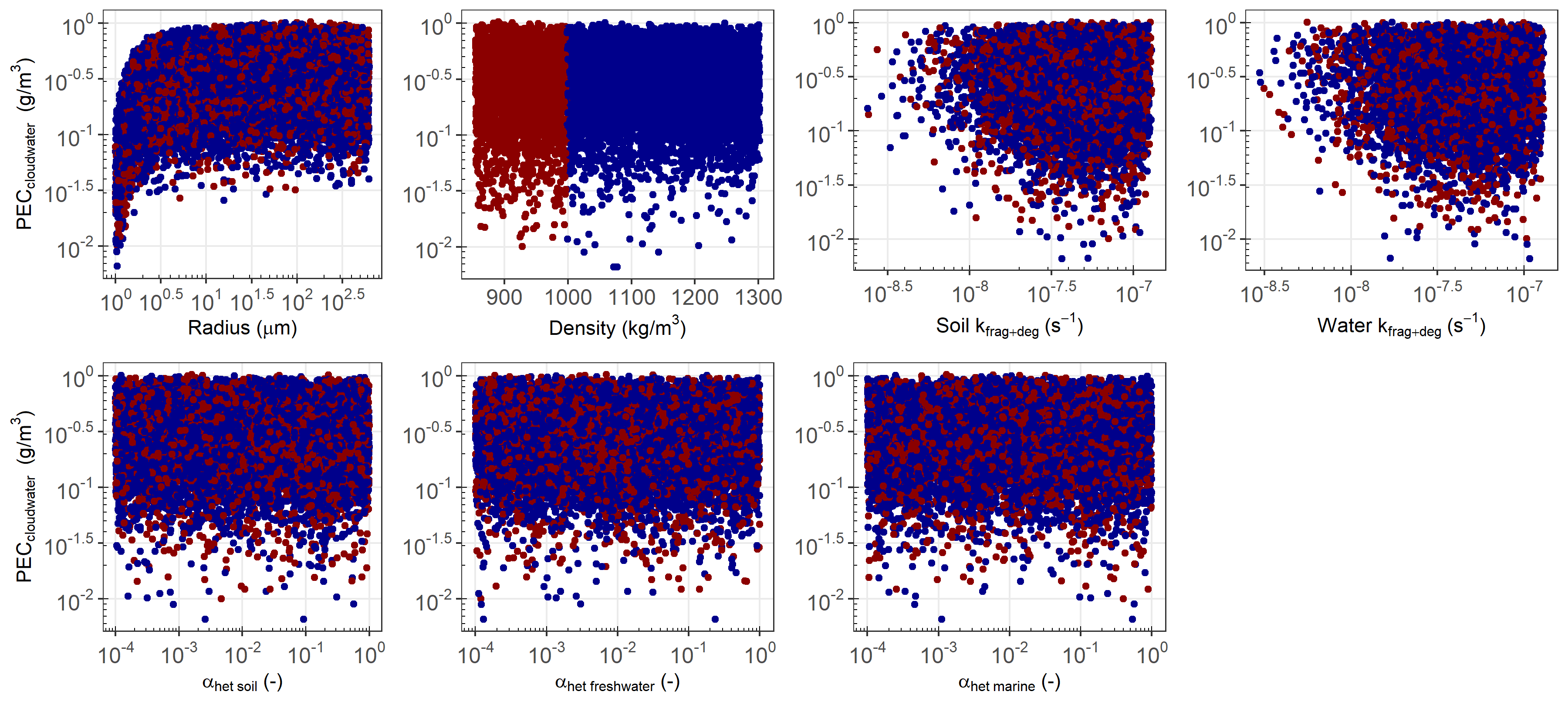

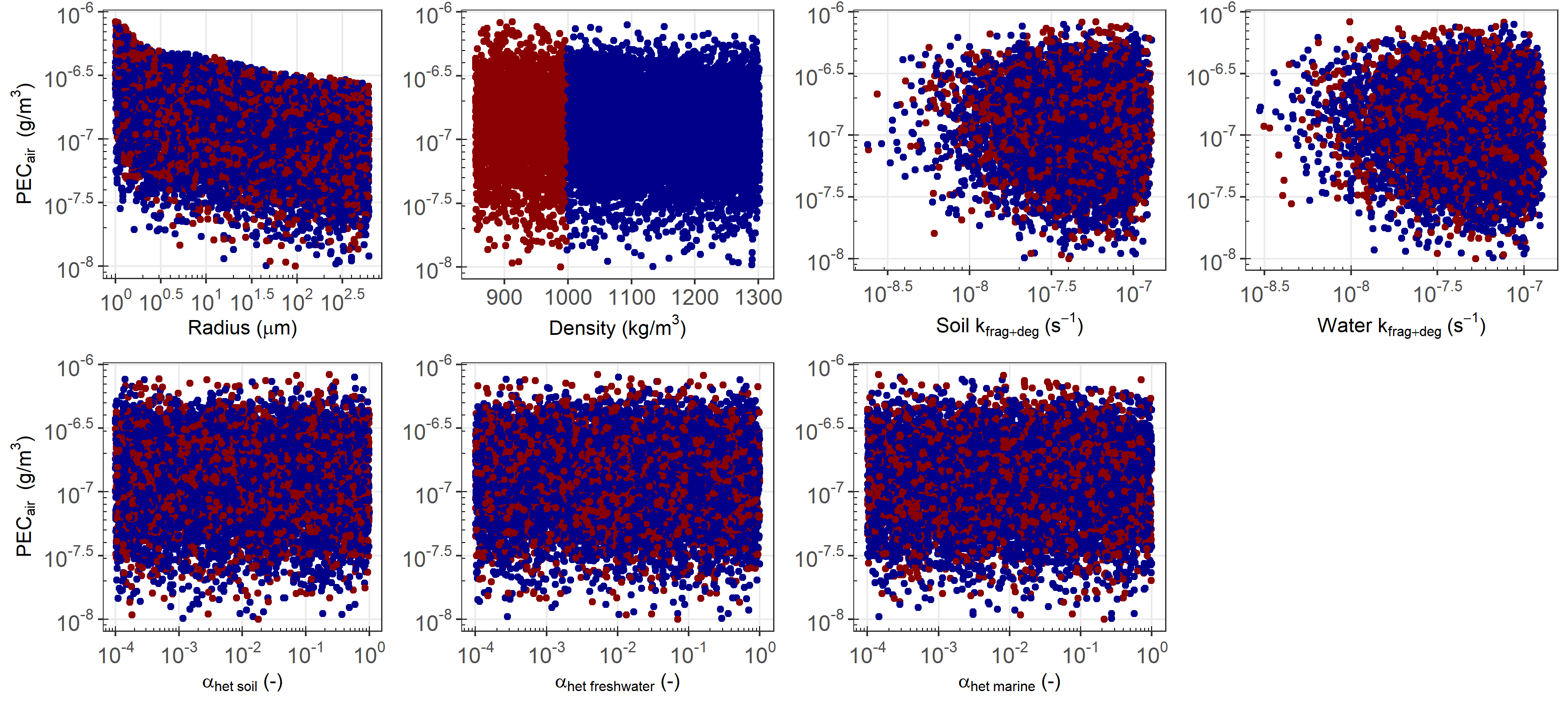


**C**

**Figure S3:** NMP Concentrations in dry air (A), in cloud water (B) and for air including cloud water (C) at regional scale.


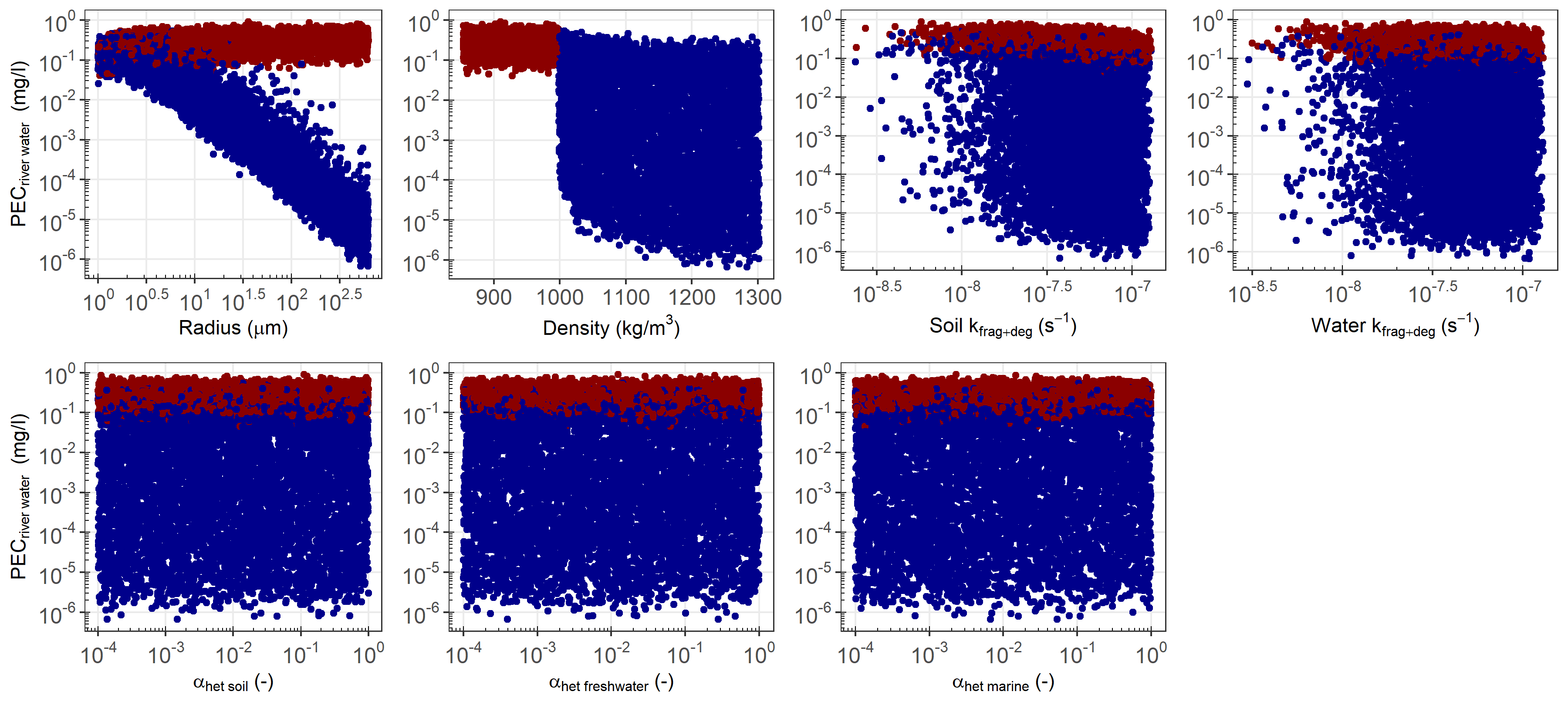


**A**


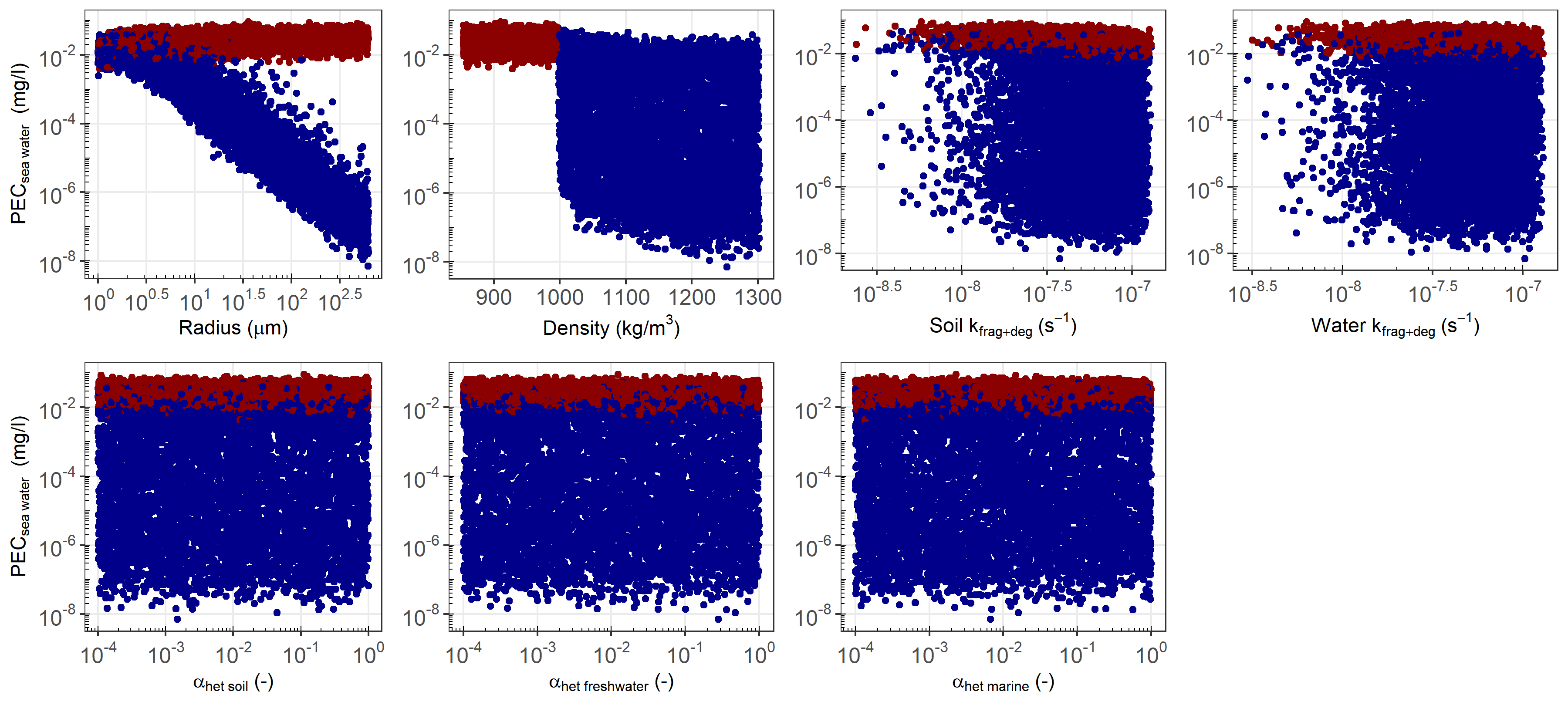


**B**

Figure S4: Concentrations of NMP in river water (A) and sea water (B) at regional scale as calculated with simplebox4Plastic.


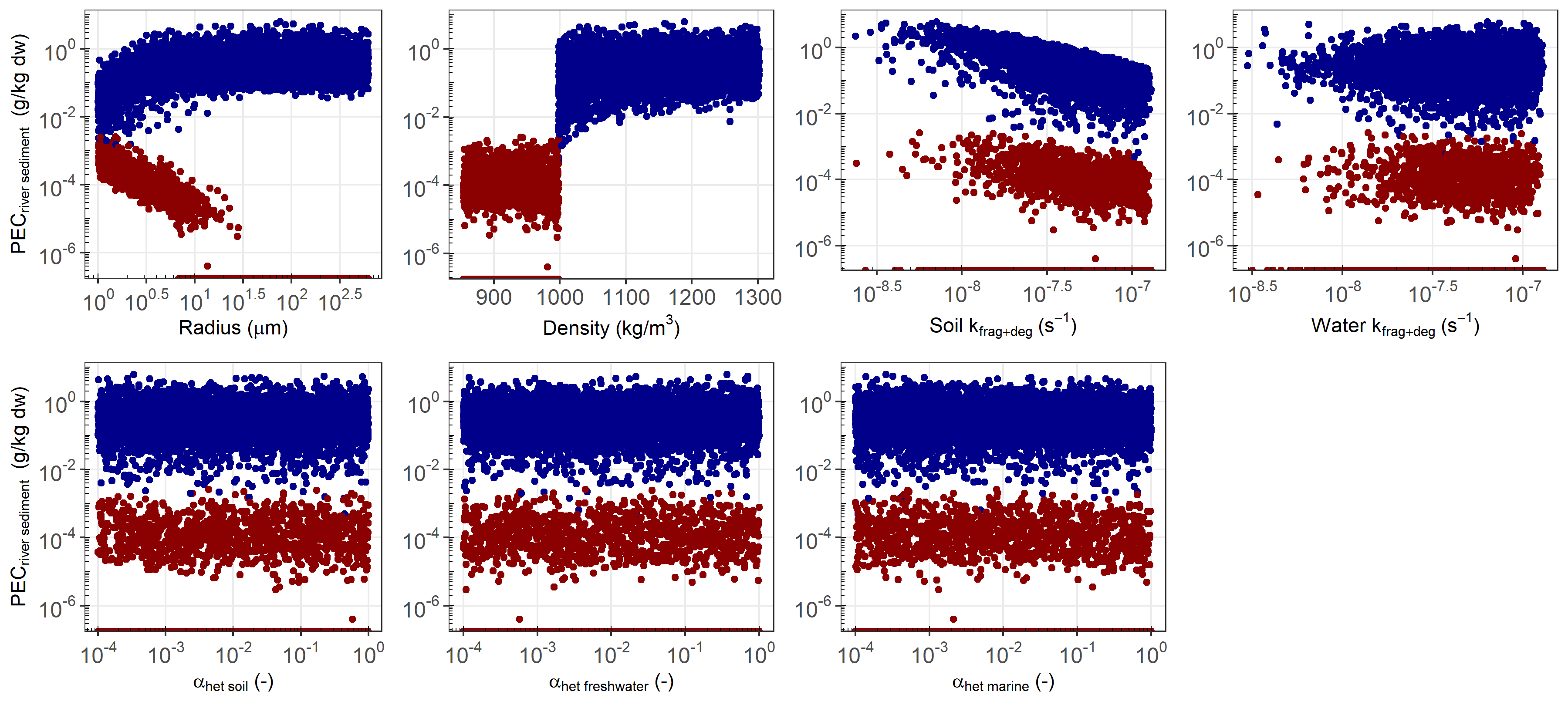


**A**


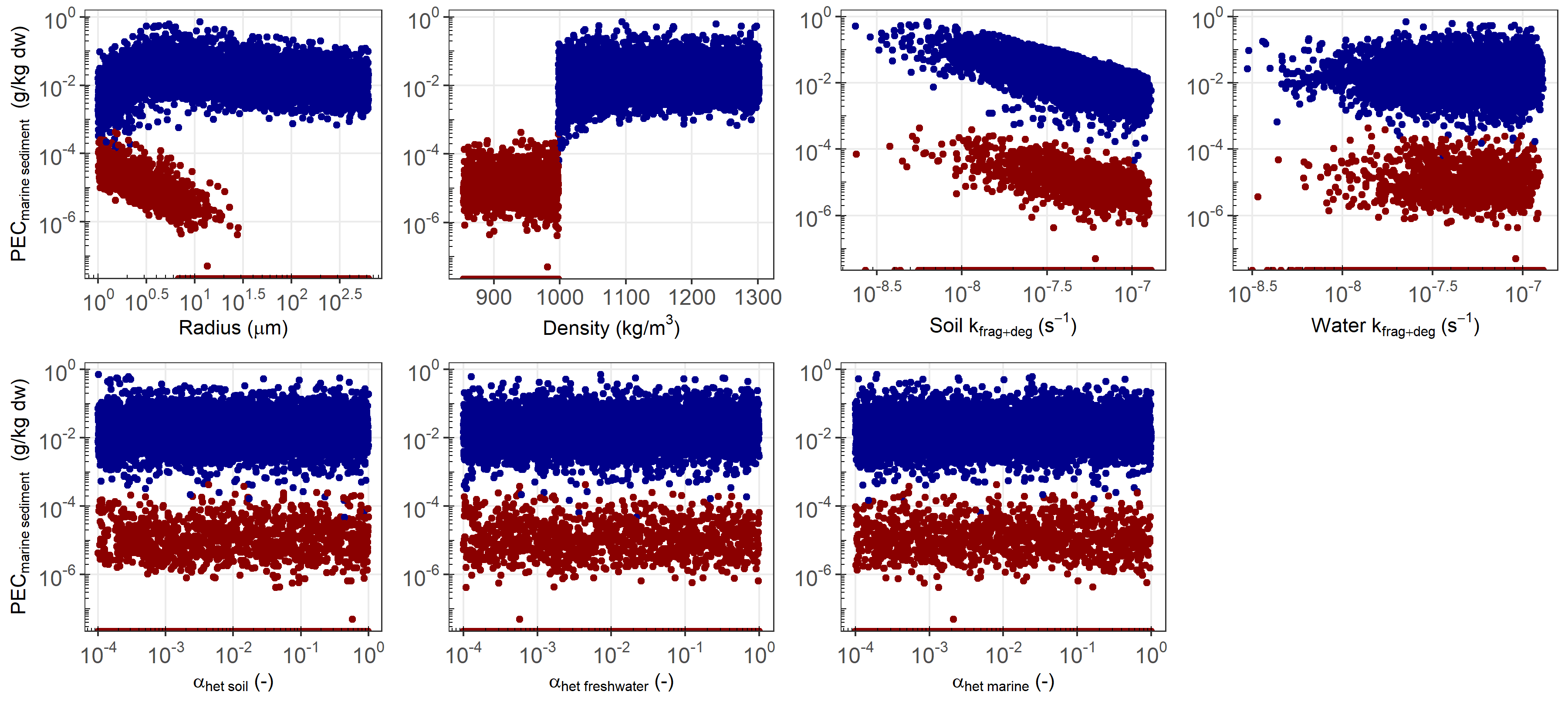


**B**

Figure S5: Concentration of microbeads in river water sediment (A), marine sediment (B) at regional scale as calculated using SimpleBox4Plastics. Note that points drawn on the x-axis are 0 concentrations.


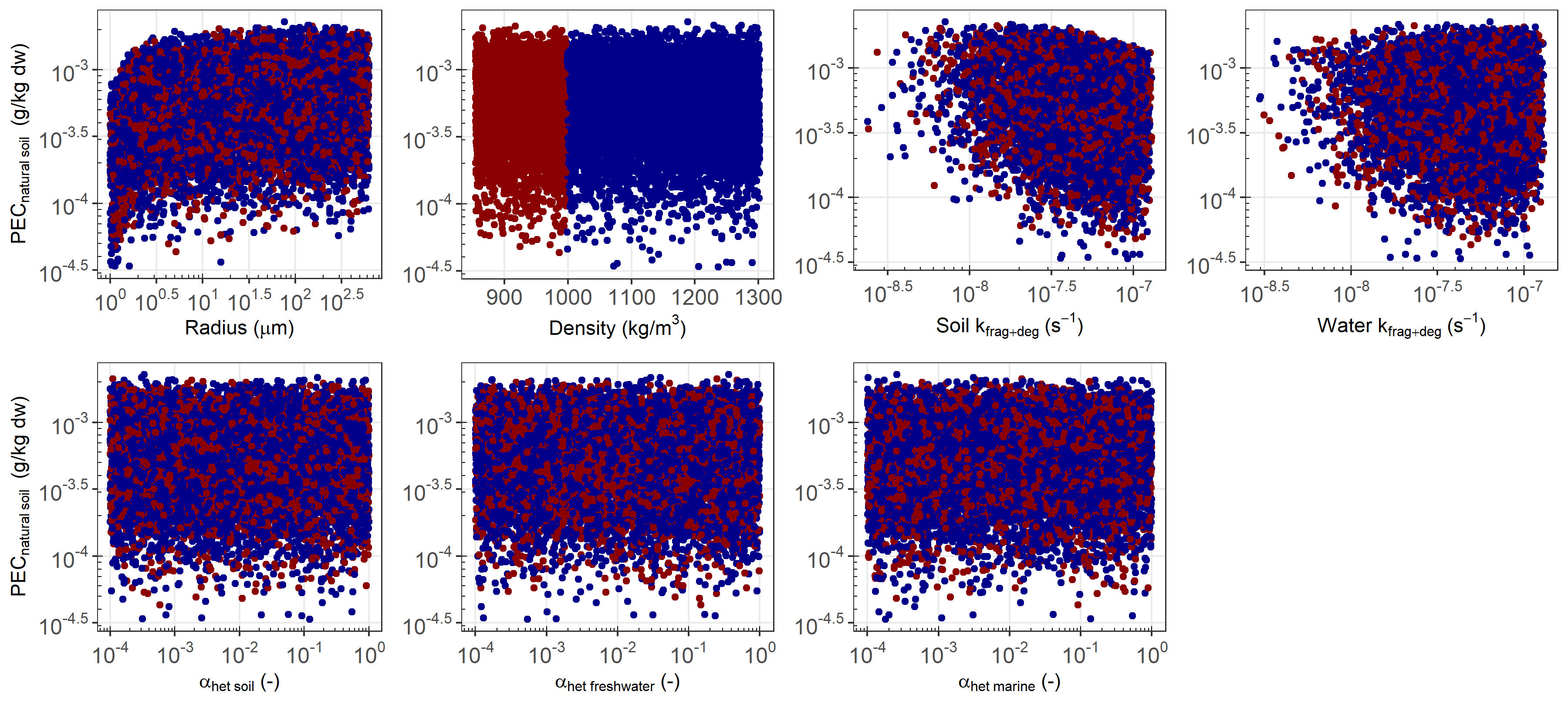


**A**


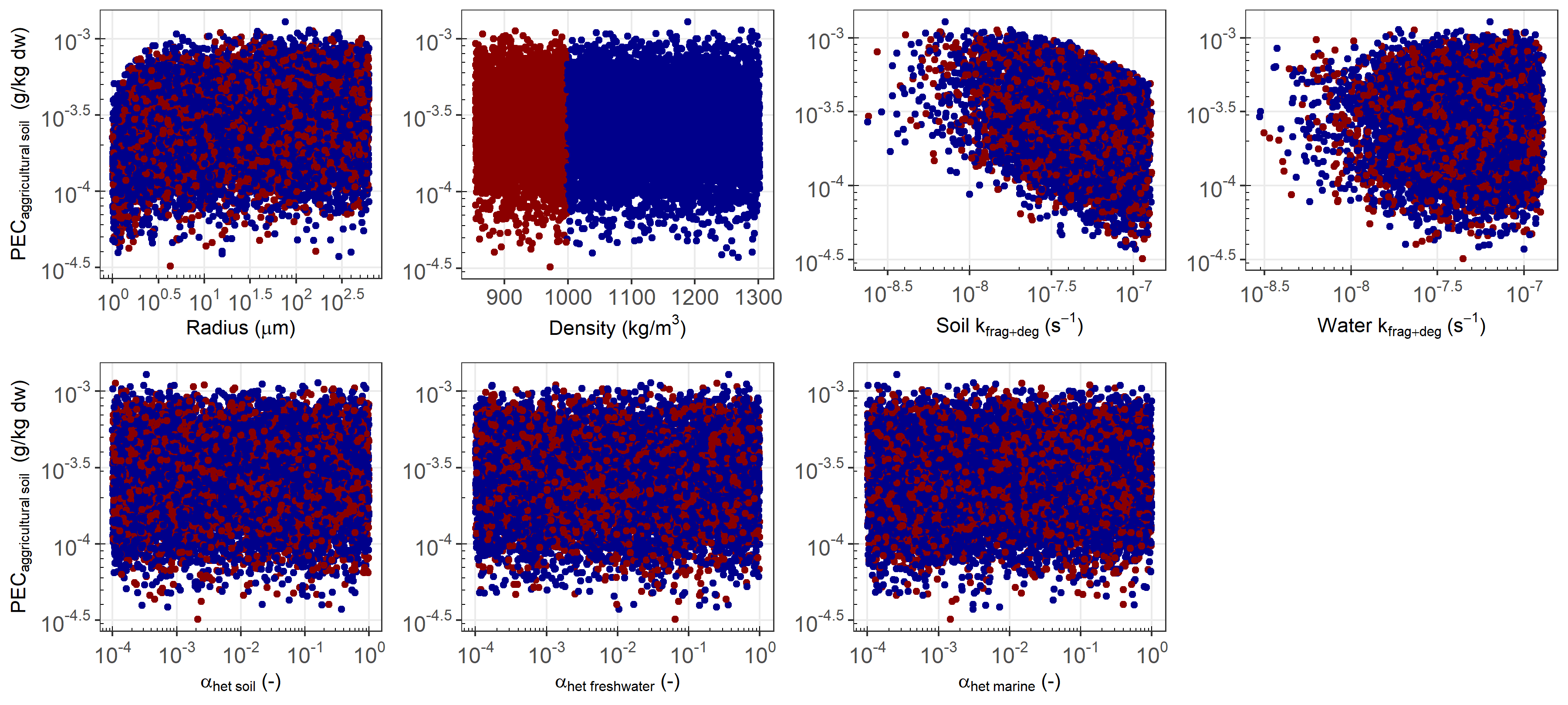


**C**

**B**


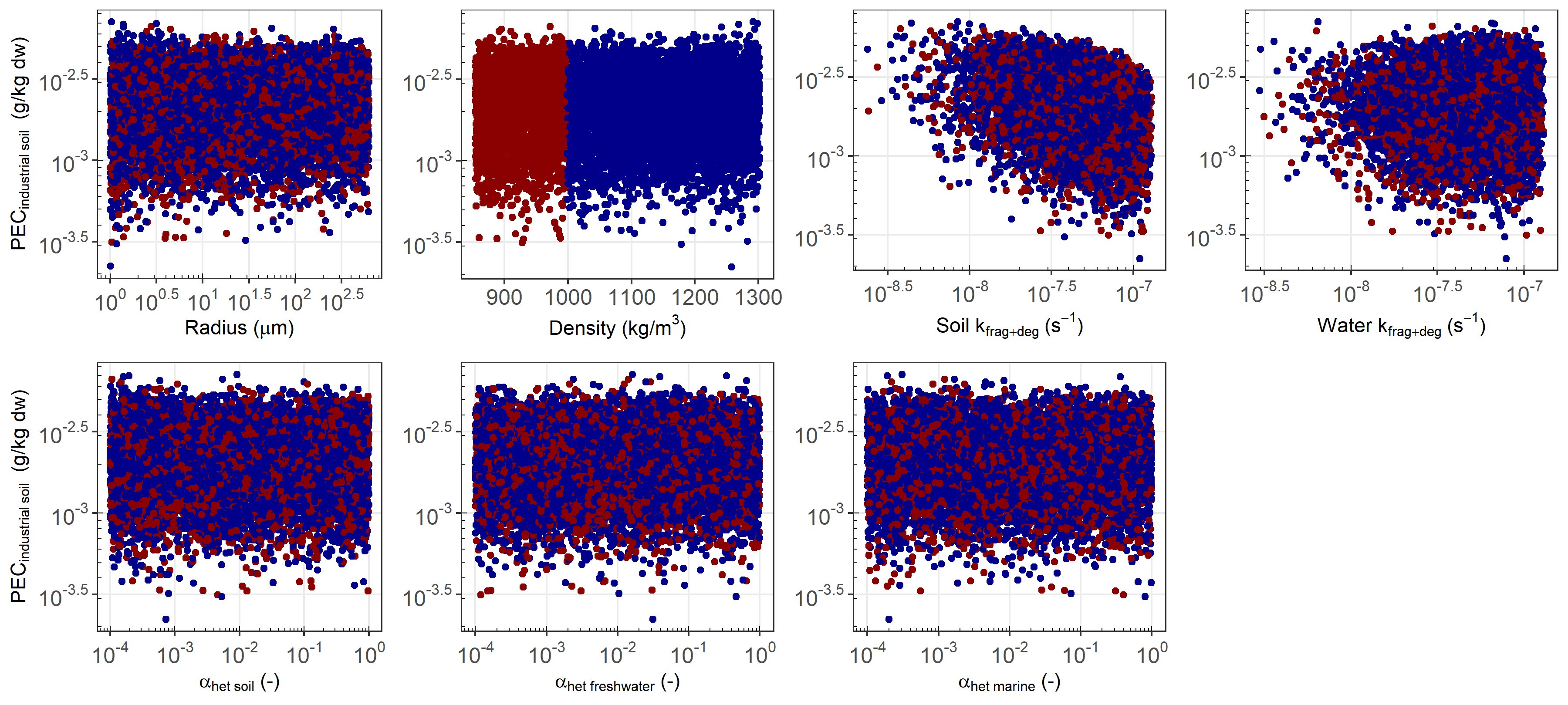


**Figure S6:** Concentration of microbeads in soil types: natural soil (A), agricultural soil (B), other/industrial soil (C) at regional scale calculated using SimpleBox4plastics.


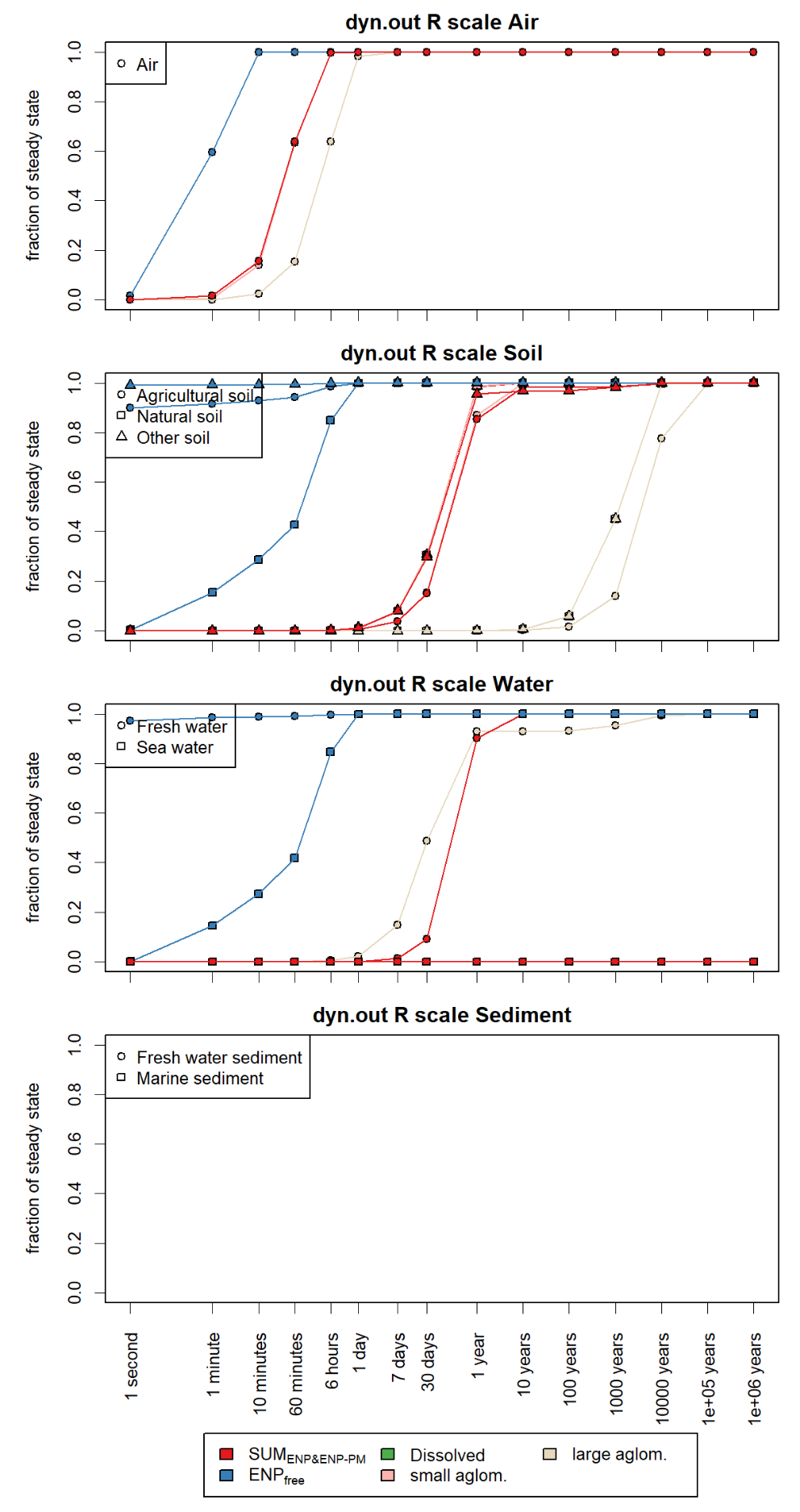


Figure S7: Time to reach steady state in air, soil, water and sediment subcompartments. Parameters for a low density scenario (fragmentation rate of 9.27E-9 s^-1^, density of 900 kg/m^3^ and radius of 25 um). Sediment (bottom panel) contains no particles due to low density floating microbeads.


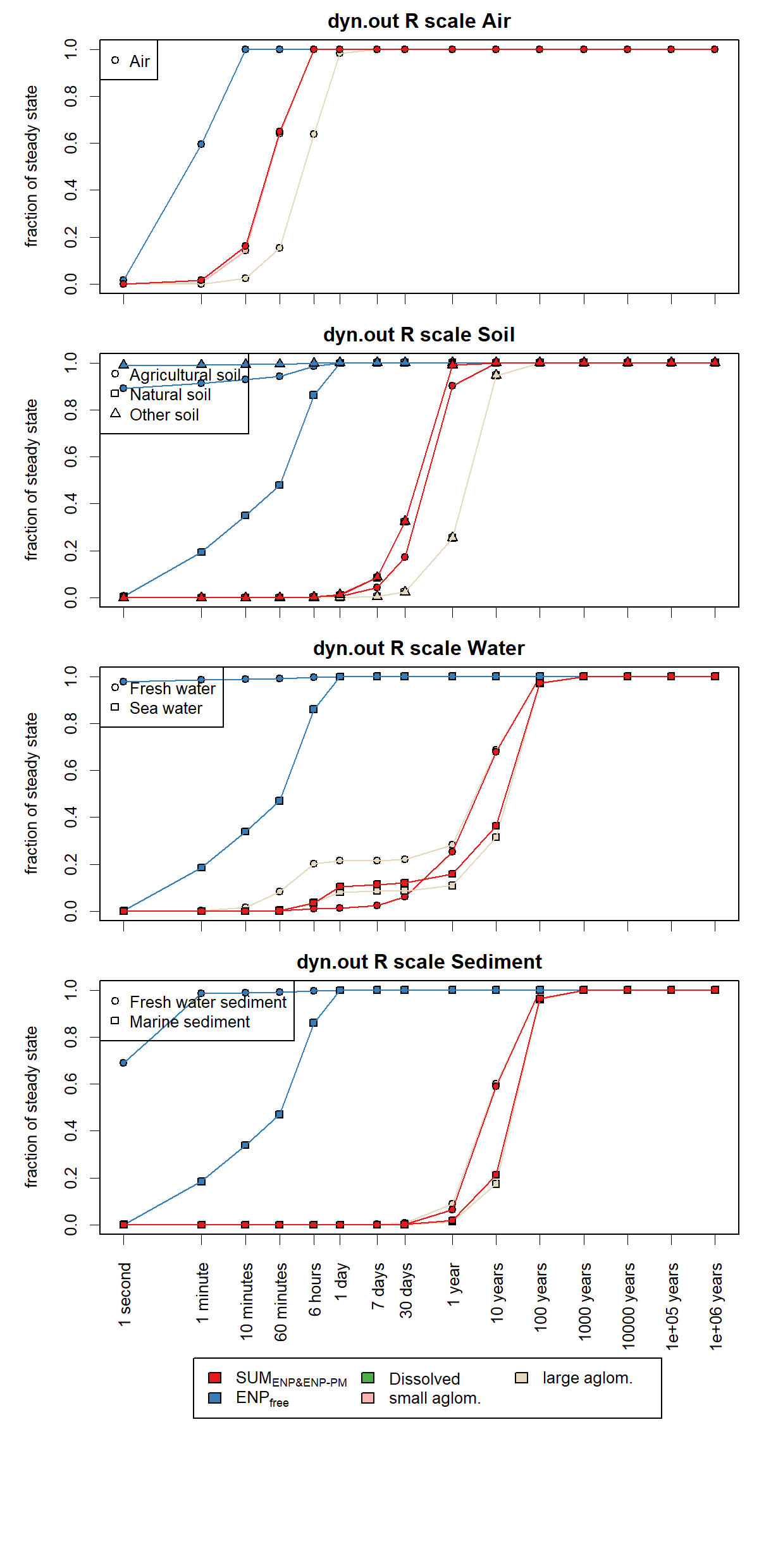


Figure S8: Time to reach steady state in air, soil, water and sediment subcompartments. Parameters for a high density scenario (fragmentation rate of 9.27E-9 s^-1^, density of 1300 kg/m^3^ and radius of 25 um).


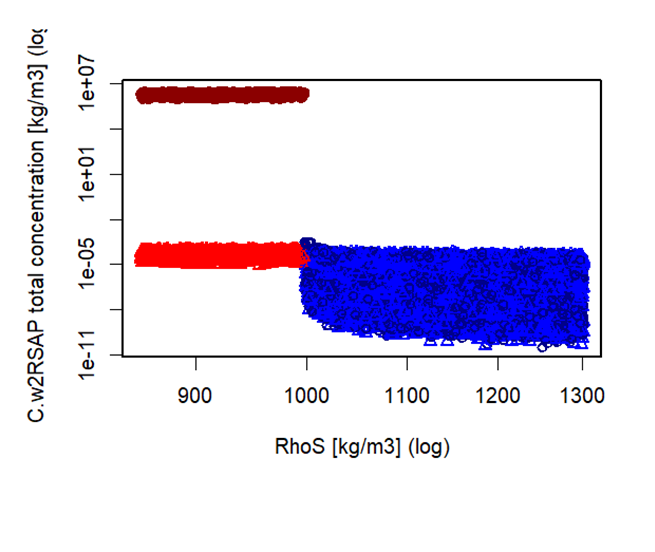


Figure S9: Concentrations of NMP in the sea water simplebox4Plastic compartment. The red color (dark and bright) and blue color (dark and bright) representing particles with density below and above water, respectively. The darker color and bright color (red and blue) represent particles without fragmentation or degradation (negigible: k_frag_deg_water = 1E-20 s^-1^) and with fragmentation (distribution Table S2), respectively.


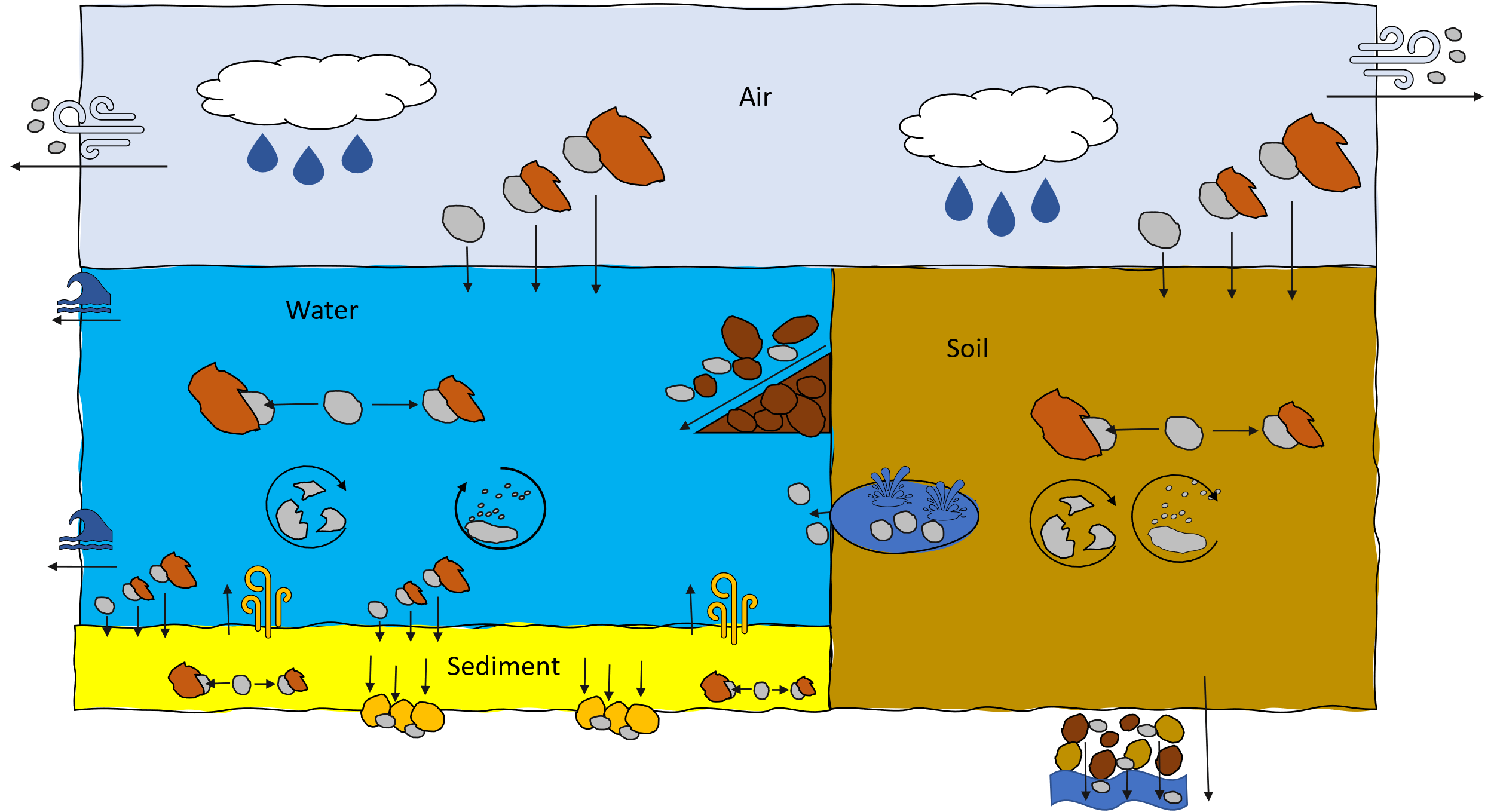


**
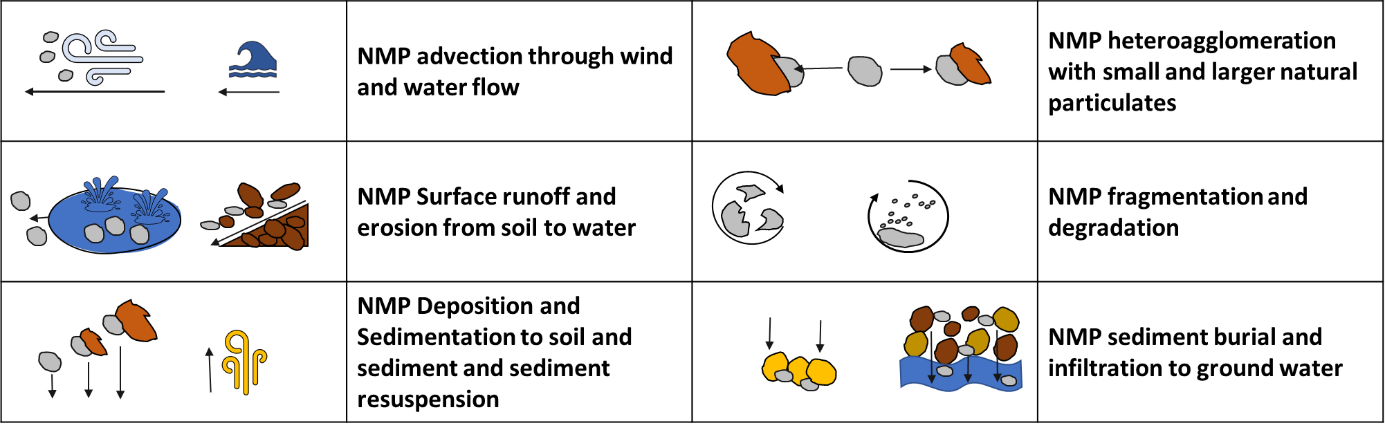
**

Figure S10: Schematic representing the processes affecting the environmental distribution of nano and microplastics (NMP) as included in SimpleBox4Plastic.

**References**

Besseling, E., Quik, J.T., Sun, M., Koelmans, A.A., 2017. Fate of nano- and microplastic in freshwater systems: A modeling study. Environ Pollut 220, 540-548.

Chamas, A., Moon, H., Zheng, J., Qiu, Y., Tabassum, T., Jang, J.H., Abu-Omar, M., Scott, S.L., Suh, S., 2020. Degradation Rates of Plastics in the Environment. ACS Sustainable Chemistry & Engineering 8, 3494-3511.

Jang, M.H., Kim, M.S., Han, M., Kwak, D.H., 2022. Experimental application of a zero-point charge based on pH as a simple indicator of microplastic particle aggregation. Chemosphere 299, 134388.

Kaandorp, M.L.A., Dijkstra, H.A., van Sebille, E., 2021. Modelling size distributions of marine plastics under the influence of continuous cascading fragmentation. Environmental Research Letters.

Koelmans, A.A., Kooi, M., Law, K.L., van Sebille, E., 2017. All is not lost: deriving a top-down mass budget of plastic at sea. Environmental Research Letters 12, 114028.

Meesters, J.A.J., Koelmans, A.A., Quik, J.T.K., Hendriks, A.J., van de Meent, D., 2014. Multimedia Modeling of Engineered Nanoparticles with SimpleBox4nano: Model Definition and Evaluation. Environmental Science & Technology 48, 5726-5736.

Scudo, A., Liebmann, B., Corden, C., Tyer, D., Kreissig, J., Warwick, O., 2017. Intentionally added microplastics in products. Amec Foster Wheeler Environment & Infrastructure UK Limited, London, United Kingdom.

Shams, M., Alam, I., Chowdhury, I., 2020. Aggregation and stability of nanoscale plastics in aquatic environment. Water Res 171, 115401.
